# Cytotoxic and Antimigratory Activities of Phenolic Compounds from *Dendrobium brymerianum*

**DOI:** 10.1155/2015/350410

**Published:** 2015-01-15

**Authors:** Pornprom Klongkumnuankarn, Kesarin Busaranon, Pithi Chanvorachote, Boonchoo Sritularak, Vichien Jongbunprasert, Kittisak Likhitwitayawuid

**Affiliations:** ^1^Department of Pharmacognosy and Pharmaceutical Botany, Faculty of Pharmaceutical Sciences, Chulalongkorn University, Bangkok 10330, Thailand; ^2^Faculty of Pharmacy, Rangsit University, Pathum Thani 12000, Thailand; ^3^Department of Pharmacology and Physiology, Faculty of Pharmaceutical Sciences, Chulalongkorn University, Bangkok 10330, Thailand; ^4^Cell-Based Drug and Health Product Development Research Unit, Faculty of Pharmaceutical Sciences, Chulalongkorn University, Bangkok 10330, Thailand

## Abstract

Chromatographic separation of a methanol extract prepared from the whole plant of *Dendrobium brymerianum* led to the isolation of eight phenolic compounds. Among the isolated compounds (**1**–**8**), moscatilin (**1**), gigantol (**3**), lusianthridin (**4**), and dendroflorin (**6**) showed appreciable cytotoxicity against human lung cancer cell lines with IC_50_ values of 196.7, 23.4, 65.0, and 125.8 *μ*g/mL, respectively, and exhibited antimigratory property at nontoxic concentrations. This study is the first report on the biological activities of this plant.

## 1. Introduction

High incidence of cancer metastasis in lung cancer makes this type of cancer among the most severe in the world [1]. Although the patients who receive appropriate treatments have approximately 15-16% of 5-year survival, the relapse of the disease cannot be ignored [2]. Metastasis of cancer cells involves the ability of the cells to migrate away from their origin. The cells migrate and invade through the basement membrane and enter the blood circulation. At their appropriate secondary sites, the cells adhere on the endothelial surface, migrate and invade out of the vessel, and establish themselves to form metastasis [3]. Currently, research on the motility of cancer cells has garnered increased attention, since the discovery of novel antimigratory compounds could lead to new cancer treatments.

Several plants in the genus* Dendrobium* (Orchidaceae) have long been used in traditional Chinese medicine as tonics [4]. Previous pharmacological studies showed that some* Dendrobium* species were potential sources of cytotoxic compounds [5]. A number of cytotoxic constituents have been reported from* Dendrobium*, for example, dengraols A and B from* D. gratiosissimum* Rchb. f. [6], erianin from* D. chrysotoxum* Lindl. [7], and moscatilin from several* Dendrobium* species [8]. The compound moscatilin is of particular interest, as it showed significant antimetastatic activity with relatively low cytotoxicity [9, 10].* Dendrobium brymerianum* Rchb. f. is a plant found in Thailand, Burma, Laos, and China [11, 12]. Recently, some phenolic compounds have been identified from this plant [12], but their cytotoxic and antimigratory activities have not been examined. As part of our continuing studies on anticancer agents from* Dendrobium* plants [13, 14], a MeOH extract obtained from the whole plant of* D. brymerianum* was evaluated and found to possess significant cytotoxicity against human lung cancer H460 cells, showing 80% inhibition at a concentration of 50 *μ*g/mL. This prompted us to investigate the plant to identify the cytotoxic components, as well as determine their antimigratory activity against cancer cells.

## 2. Material and Methods

### 2.1. General

Mass spectra were recorded on a UPLC mass spectrophotometer (Waters 2996-2695, ESI-MS). NMR spectra were recorded on a Bruker Avance DPX-300 FT-NMR spectrometer or a Varian Unity INOVA-500 NMR spectrometer. Optical rotation was measured on a Perkin-Elmer 341 polarimeter. Vacuum-liquid column chromatography (VLC) and column chromatography (CC) were performed on silica gel 60 (Merck, Kieselgel 60, 70–320 mesh), silica gel 60 (Merck, Kieselgel 60, 230–400 mesh), and Sephadex LH-20 (25–100 *μ*m, Pharmacia Fine Chemical Co. Ltd.).

### 2.2. Plant Material

Sample of* D. brymerianum* was purchased from Jatujak market, Bangkok, Thailand, in September 2012 and identified by Professor Thatree Phadungcharoen (Department of Pharmacognosy and Pharmaceutical Botany, Faculty of Pharmaceutical Sciences, Chulalongkorn University). A voucher specimen (BS-DB-092555) is deposited at the Department of Pharmacognosy and Pharmaceutical Botany, Faculty of Pharmaceutical Sciences, Chulalongkorn University, Bangkok, Thailand.

### 2.3. Extraction and Isolation

The dried and powdered whole plant (3 kg) was macerated with MeOH (3 × 10 L) to give a MeOH extract (100 g) after removal of the solvent. This material was fractionated by vacuum-liquid chromatography (VLC) on silica gel (*n*-hexane-EtOAc gradient) to give 6 fractions (A–F). Fraction F (16.0 g) was separated by column chromatography (CC) over silica gel and eluted with* n*-hexane-EtOAc gradient, to give 11 fractions (FI–FXI). Fraction FV (2.2 g) was separated on Sephadex LH20 (acetone) to give 8 fractions (FV1–FV8). Compounds** 1** (100 mg) and** 2** (47 mg) were obtained from fraction FV4 (390 mg) after purification on Sephadex LH 20 (acetone). Fraction FV5 (1.0 g) was further purified on Sephadex LH 20 (acetone) to furnish compound** 3** (50 mg). Purification of fraction FV6 (108 mg) on Sephadex LH 20 (acetone) gave compound** 4** (5 mg). Fraction FVI (4.8 g) was separated by CC over silica gel (CH_2_Cl_2_-EtOAc gradient) to yield 7 fractions (FVI1–FVI7). Fraction FVI4 (1.7 g) was separated on Sephadex LH 20 (acetone) and further purified by CC (silica gel; CH_2_Cl_2_-EtOAc, 9 : 1) to afford compounds** 5** (6 mg) and** 6** (74 mg), respectively. Fraction FVI6 (93 mg) was separated by CC (silica gel; CH_2_Cl_2_-EtOAc, gradient) to give compounds** 7** (6 mg) and** 8** (4 mg), respectively.

*Moscatilin (* ***1***). Brown amorphous solid; C_17_H_20_O_5_; ESI-MS* m/z* 305 [M+H]^+^; ^1^H NMR (500 MHz, acetone-*d*_6_) *δ*: 2.78 (4H, m, H_2_-*α*, H_2_-*α*′), 3.75 (6H, s, MeO-3, MeO-5), 3.76 (3H, s, MeO-3′), 6.48 (2H, s, H-2,6), 6.64 (1H, dd, *J* = 8.0, 2.0 Hz, H-6′), 6.75 (1H, d, *J* = 8.0 Hz, H-5′), 6.78 (1H, d, *J* = 2.0 Hz, H-2′); ^13^C NMR (125 MHz, acetone-*d*_6_) *δ*: 38.3 (C-*α*′), 38.8 (C-*α*), 56.1 (MeO-3′), 56.5 (MeO-3, MeO-5), 106.7 (C-2, C-6), 112.9 (C-2′), 115.4 (C-5′), 121.6 (C-6′), 133.1 (C-1), 134.1 (C-1′), 134.8 (C-4), 145.3 (C-4′), 147.9 (C-3′), 148.3 (C-3,5). 

*Flavanthrinin (* ***2*** *).* Brown amorphous solid; C_15_H_12_O_3_; ESI-MS* m/z* 241 [M+H]^+^; ^1^H NMR (500 MHz, acetone-*d*_6_) *δ*: 4.15 (3H, s, MeO-4), 6.98 (1H, d, *J* = 2.5 Hz, H-3), 7.06 (1H, d, *J* = 2.5 Hz, H-1), 7.08 (1H, dd, *J* = 7.5, 2.5 Hz, H-6), 7.40 (1H, d, *J* = 2.5 Hz, H-8), 7.42 (1H, d, *J* = 7.5 Hz, H-5), 7.49 (1H, d, *J* = 9.0 Hz, H-10), 7.62 (1H, d, *J* = 9.0 Hz, H-9); ^13^C NMR (125 MHz, acetone-*d*_6_) *δ*: 58.5 (MeO-4), 102.5 (C-3), 107.7 (C-1), 114.0 (C-4a), 116.9 (C-6), 119.9 (C-4b), 121.0 (C-8), 126.9 (C-10), 127.4 (C-5), 129.7 (C-9), 134.9 (C-8a), 137.0 (C-10a), 155.2 (C-7), 156.3 (C-2), 157.3 (C-4). 

*Gigantol (* ***3*** *).* Brown amorphous solid; C_16_H_18_O_4_; ESI-MS* m/z* 275 [M+H]^+^; ^1^H NMR (500 MHz, acetone-*d*_6_) *δ*: 2.78 (4H, m, H_2_-*α*, H_2_-*α*′), 3.69 (3H, s, MeO-3′), 3.78 (3H, s, MeO-3), 6.22 (1H, t, *J* = 2.0 Hz, H-2), 6.28 (1H, t, *J* = 2.0 Hz, H-4), 6.30 (1H, t, *J* = 2.0 Hz, H-6), 6.64 (1H, dd, *J* = 8.0, 1.5 Hz, H-6′), 6.69 (1H, d, *J* = 8.0 Hz, H-5′), 6.79 (1H, d, *J* = 1.5 Hz, H-2′); ^13^C NMR (125 MHz, acetone-*d*_6_) *δ*: 37.9 (C-*α*′), 39.0 (C-*α*), 55.2 (MeO-3), 56.0 (MeO-3′), 99.6 (C-4), 106.2 (C-6), 108.8 (C-2), 112.8 (C-5′), 115.4 (C-2′), 121.5 (C-6′), 134.0 (C-1′), 145.1 (C-4′), 145.4 (C-1), 147.9 (C-3′), 159.1 (C-3), 161.7 (C-5). 

*Lusianthridin (* ***4*** *).* Brown amorphous solid; C_15_H_14_O_3_; ESI-MS* m/z* 243 [M+H]^+^; ^1^H NMR (500 MHz, acetone-*d*_6_) *δ*: 3.72 (3H, s, MeO-2), 6.36 (1H, d, *J* = 1.5 Hz, H-1), 6.42 (1H, d, *J* = 1.5 Hz, H-3), 6.67 (1H, dd, *J* = 9.0, 2.5 Hz, H-6), 6.70 (1H, d, *J* = 2.5 Hz, H-8), 8.22 (1H, d, *J* = 9.0 Hz, H-5); ^13^C NMR (125 MHz, acetone-*d*_6_) *δ*: 30.6 (C-9), 31.4 (C-10), 55.2 (MeO-2), 101.5 (C-3), 105.8 (C-1), 113.4 (C-6), 114.9 (C-8), 115.7 (C-4a), 125.8 (C-4b), 129.8 (C-5), 139.7 (C-8a), 141.3 (C-10a), 155.8 (C-4), 155.9 (C-7), 159.2 (C-2).

*Nobilone (* ***5*** *).* Red amorphous solid; C_14_H_10_O_4_; ESI-MS* m/z* 243 [M+H]^+^; ^1^H NMR (500 MHz, acetone-*d*_6_) *δ*: 4.13 (3H, s, MeO-4), 6.78 (1H, d, *J* = 2.0 Hz, H-3), 6.80 (1H, d, *J* = 2.0 Hz, H-1), 6.93 (1H, dd, *J* = 7.5, 1.5 Hz, H-6), 7.10 (1H, d, *J* = 1.5 Hz, H-8), 7.13 (1H, d, *J* = 7.5 Hz, H-5); ^13^C NMR (125 MHz, acetone-*d*_6_) *δ*: 57.5 (MeO-4), 105.9 (C-1), 106.2 (C-3), 116.7 (C-8), 122.6 (C-4a), 125.0 (C-6), 128.0 (C-4b), 130.2 (C-5), 135.8 (C-8a), 137.2 (C-9a), 151.6 (C-7), 153.5 (C-4), 160.9 (C-2), 193.2 (C-9).

*Dendroflorin (* ***6*** *).* Red amorphous solid; C_14_H_10_O_5_; ESI-MS* m/z* 259 [M+H]^+^; ^1^H NMR (500 MHz, acetone-*d*_6_) *δ*: 4.10 (3H, s, MeO-4), 6.58 (1H, d, *J* = 9.0 Hz, H-7), 6.76 (1H, d, *J* = 1.6 Hz, H-3), 6.79 (1H, d, *J* = 1.6 Hz, H-1), 6.87 (1H, d, *J* = 9.0 Hz, H-6); ^13^C NMR (125 MHz, acetone-*d*_6_) *δ*: 57.4 (MeO-4), 105.6 (C-1), 106.1 (C-3), 117.4 (C-8a), 119.7 (C-7), 122.4 (C-4a), 124.3 (C-4b), 128.9 (C-6), 137.4 (C-9a), 145.1 (C-5), 152.8 (C-8), 154.1 (C-4), 160.9 (C-2), 195.3 (C-9).

*Denchrysan B (* ***7*** *).* Red amorphous solid; C_14_H_12_O_4_; ESI-MS* m/z* 245 [M+H]^+^; [*α*]_*D*_^20^  −7.9 (*c* = 0.1, MeOH); ^1^H NMR (300 MHz, acetone-*d*_6_) *δ*: 4.06 (3H, s, MeO-4), 5.37 (1H, br s, H-9), 6.59 (1H, br s, H-3), 6.73 (1H, dd, *J* = 8.1, 1.5 Hz, H-6), 6.83 (1H, br s, H-1), 7.03 (1H, m, H-8), 7.07 (1H, m, H-7); ^13^C NMR (75 MHz, acetone-*d*_6_) *δ*: 57.0 (MeO-4), 75.4 (C-9), 100.3 (C-3), 107.2 (C-1), 116.9 (C-6), 117.0 (C-8), 118.9 (C-4a), 124.7 (C-4b), 128.4 (C-7), 148.5 (C-8a), 150.8 (C-9a), 151.4 (C-5), 153.0 (C-4), 159.6 (C-2).

*Tristin (* ***8*** *).* Brown amorphous solid; C_15_H_16_O_4_; ESI-MS* m/z* 261 [M+H]^+^; ^1^H NMR (300 MHz, acetone-*d*_6_) *δ*: 2.74 (4H, m, H_2_-*α*, H_2_-*α*′), 3.78 (3H, s, MeO-3′), 6.17 (1H, d, *J* = 1.8 Hz, H-4), 6.20 (2H, d, *J* = 1.8 Hz, H-2, H-6), 6.63 (1H, dd, *J* = 7.8, 1.5 Hz, H-6′), 6.69 (1H, d, *J* = 7.8 Hz, H-5′), 6.79 (1H, d, *J* = 1.5 Hz, H-2′); ^13^C NMR (75 MHz, acetone-*d*_6_) *δ*: 37.9 (C-*α*′), 38.9 (C-*α*), 56.1 (MeO-3′), 101.0 (C-4), 107.8 (C-2, C-6), 112.8 (C-2′), 115.5 (C-5′), 121.5 (C-6′), 134.1 (C-1′), 145.1 (C-4′), 145.5 (C-1), 148.0 (C-3′), 159.2 (C-3, C-5).

### 2.4. Cells and Reagents

Lung cancer H460 cells were obtained from the American Type Culture Collection (Manassas, VA). Cells were cultured in RPMI 1640 containing 5% fetal bovine serum, 2 mM l-glutamine, and 100 units/mL penicillin/streptomycin in a 5% CO_2_ environment at 37°C. 3-(4,5-Dimethylthiazol-2-yl)-2,5-diphenyltetrazolium bromide (MTT) and other chemicals were obtained from Sigma Chemical, Inc. (St. Louis, MO).

### 2.5. Cell Viability Assay

To determine cytotoxicity, cell viability was determined by modified MTT assay [15] which measured cellular capacity to reduce MTT (yellow) to purple formazan crystal by mitochondrial dehydrogenase. The cells were seeded at a density of 1 × 10^4^ cells/well in a 96-well plate and allowed to attach for 12 h, treated with different concentrations of compounds in DMSO (with the final concentration of DMSO less than 0.1% in each well) for 24 h, and then incubated with 100 *μ*L of 500 *μ*g/mL MTT solution for 4 h at 37°C. Then, MTT solution was removed, and 100 *μ*L of 99.9% DMSO was added to dissolve the formazan crystal. The intensity of formazan product was measured at 570 nm using a microplate reader. All analyses were performed in at least three independent replicate cultures. The cell viability was calculated as follows:

(1) $$\begin{array}{c} \text{Cell}\;\text{viability}\;\left(\%\right)=\frac{{\text{A}}_{570}\;\text{of}\;\text{treatment}}{{\text{A}}_{570}\;\text{of}\;\text{untreated}\;\text{control}}\times 100. \end{array}$$

### 2.6. Assay for Antimigratory Activity

Migratory activity of the cancer cells was evaluated by wound-healing assay [16]. Briefly, a monolayer of cells at a density of 1.5 × 10^4^ cells/well was cultured in 96-well plates, and a wound space was created by a 1 mm width tip. After rinsing with PBS, the cell monolayers were treated with nontoxic concentration of compounds (0.1 *μ*g/mL) and allowed to migrate for 0–48 h. Micrographs were taken under a phase-contrast microscope (×100; Olympus IX51 with DP70), and wound spaces were measured from 10 random fields of view using Olympus DP Controller software. Quantitative analysis of cell migration was performed by using an average wound space from random fields of view, and the relative migration was calculated.

### 2.7. Data/Statistical Analysis

All results from three or more independent experiments were expressed as means ± SD. Statistical differences between means were determined using the analysis of variance (ANOVA) and the post hoc test. A *P* value of less than 0.05 (*P* < 0.05) was considered statistically significant. The IC_50_ value was obtained using the GraphPad Prism software.

### 2.8. Determination of Total Phenolic Content

The total phenolic content (TPC) was determined by Folin-Ciocalteu method [17]. Assays were conducted in a 96-well microtiter plate. Briefly, 20 *μ*L of the plant extract solution (0.5 mg/mL in ethanol) was added to 1 : 10 diluted Folin-Ciocalteu reagent (100 *μ*L). After 5 minutes, 100 *μ*L of Na_2_CO_3_ (75 g/100 mL in deionized water) was added. After incubation at room temperature for 1 hr, the absorbance was measured at 765 nm using a Perkin-Elmer Victor3 1420 multilabel counter. Gallic acid (Sigma) was used as a reference standard, and the total phenolic content was expressed as mg of gallic acid equivalent (GAE)/g dried plant material.

## 3. Results and Discussion

**Figure 1:**
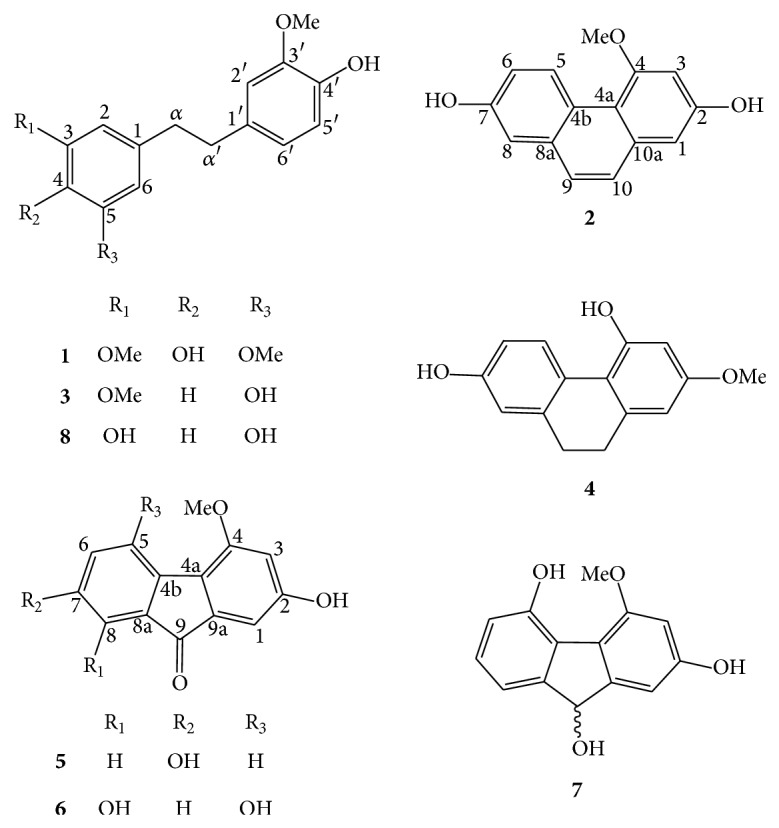
Structures of compounds** 1**–**8** from* Dendrobium brymerianum.*

The total phenolic content of the dried plant material was found to be 1.13 mg GAE/g, which was in the same level as that of* Dendrobium nobile* [18]. Chromatographic separation of this MeOH extract resulted in the isolation of eight phenolic compounds which included moscatilin (**1**) [19], flavanthrinin (**2**) [20], gigantol (**3**) [21], lusianthridin (**4**) [22], nobilone (**5**) [23], dendroflorin (**6**) [24], denchrysan B (**7**) [25], and tristin (**8**) [21]. The structures of these isolates (Figure 1) were determined through analysis of their ^1^H NMR, ^13^C NMR, and MS data in comparison with previously reported values. Compounds** 1**,** 2**,** 4**,** 5**,** 7**, and** 8** were not found in the previous study [12].

**Figure 2:**
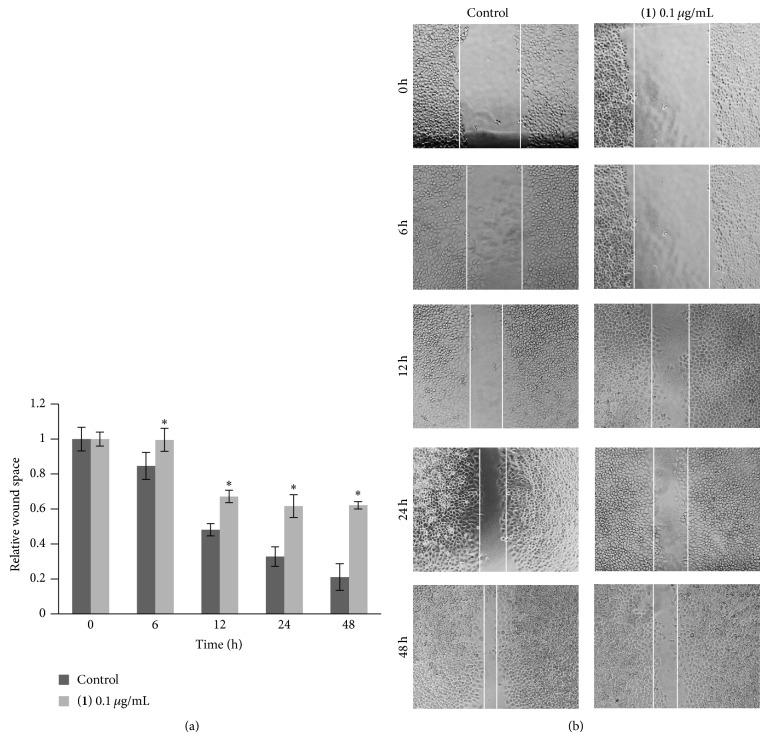
Effect of moscatilin (**1**) on H460 cell migration. (a) Confluent monolayer of H460 cells was wounded using a 1 mm width tip and treated with moscatilin (**1**) at 0.1 *μ*g/mL or without it for various times (0–48 h). Wound space was analyzed and represented as migration level relative to the change of those in untreated cells. Data represent the mean ± SD (*n* = 3). ^*^ *P* < 0.05 versus untreated control cells. (b) Wound space was visualized under a phase-contrast microscope at the indicated times.

**Figure 3:**
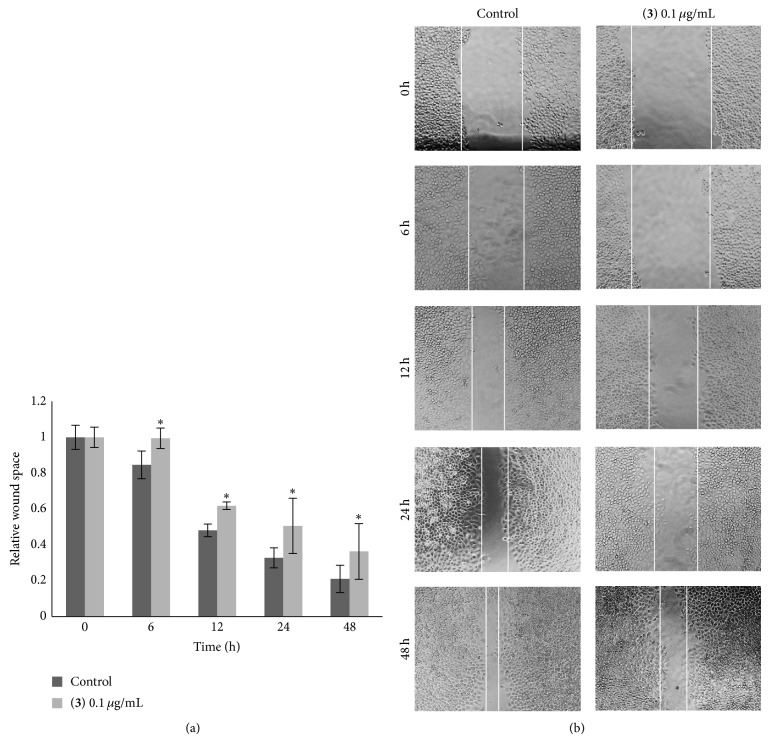
Effect of gigantol (**3**) on H460 cell migration. (a) The cells were wounded and treated with gigantol (**3**) at 0.1 *μ*g/mL or without it for various times (0–48 h). Wound space was analyzed and represented as migration level relative to the change of those in untreated cells. Data represent the mean ± SD (*n* = 3). ^*^ *P* < 0.05 versus untreated control cells. (b) Wound space was visualized under a phase-contrast microscope at the indicated times.

**Figure 4:**
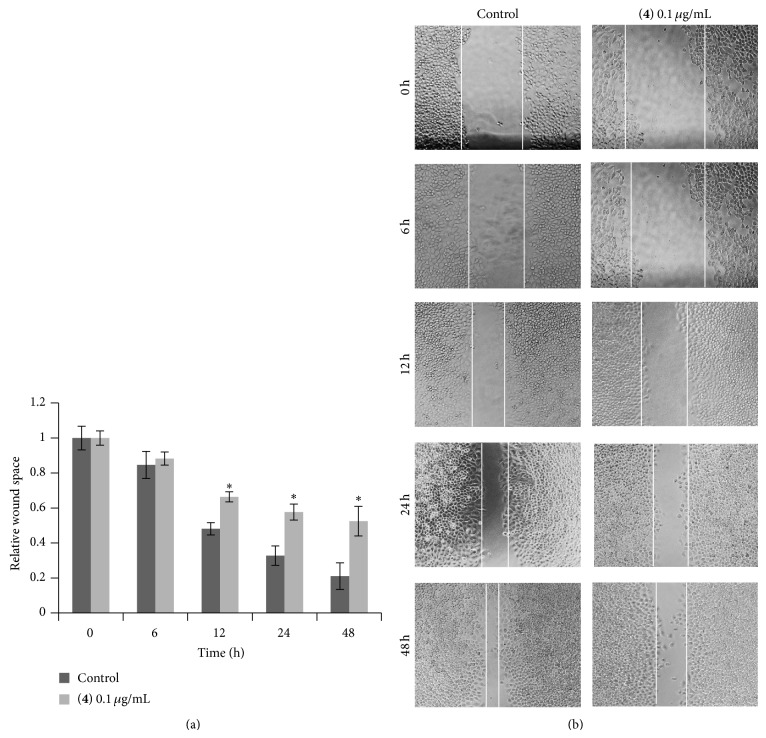
Effect of lusianthridin (**4**) on H460 cell migration. (a) Effect of lusianthridin (**4**) at 0.1 *μ*g/mL on migration of H460 cells was tested by migratory assay. Wound space was analyzed and represented as migration level relative to the change of those in untreated cells. Data represent the mean ± SD (*n* = 3). ^*^ *P* < 0.05 versus untreated control cells. (b) Wound space was visualized under a phase-contrast microscope at the indicated times.

**Figure 5:**
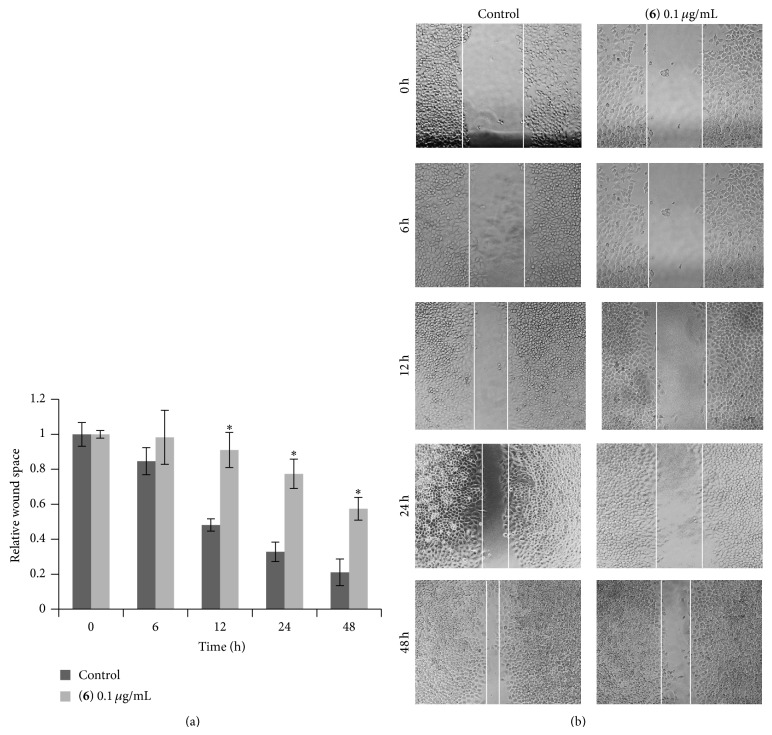
Effect of dendroflorin (**6**) on H460 cell migration. (a) H460 cells were scratched by a 1 mm width tip and treated with dendroflorin (**6**) at 0.1 *μ*g/mL or without it for various times (0–48 h). Wound space was analyzed and represented as migration level relative to the change of those in untreated cells. Data represent the mean ± SD (*n* = 3). ^*^ *P* < 0.05 versus untreated control cells. (b) Wound space was visualized under a phase-contrast microscope at the indicated times.

**Figure 6:**
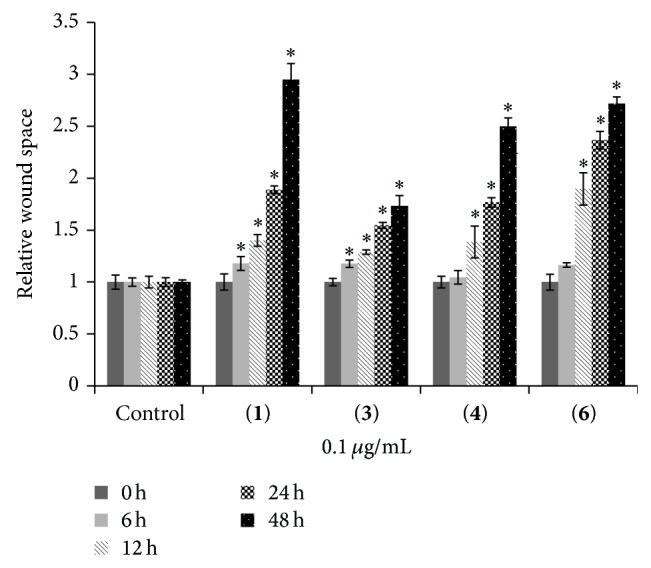
The relative wound space was analyzed by comparison of the relative change in wound space of the treated groups and that of the untreated control. Data represent the mean ± SD (*n* = 3). ^*^ *P* < 0.05 versus untreated control cells.

Compounds** 1**–**8** were evaluated for cytotoxicity against human lung cancer H460 cells using established protocols [15], and the results are summarized in Table 1. It can be seen that, apart from moscatilin (**1**) which has been earlier reported for cytotoxic and antimigratory effects [9], only gigantol (**3**), lusianthridin (**4**), and dendroflorin (**6**) exhibited appreciable cytotoxic properties, whereas the others were inactive (IC_50_ > 200 *μ*g/mL). From these preliminary limited data it could be speculated that the stronger cytotoxicity of the extract, as compared with the individual components, was probably due to the additive or synergistic effects from the eight phenolic (**1**–**8**) and/or other unisolated compounds. Compounds** 3**,** 4**, and** 6** were further investigated for antimigratory potential in comparison with** 1**, using a modified method of the wound-healing assay [16]. In a previous report, moscatilin (**1**) showed inhibition against the migration of breast MDA-MB-231 cells [10] and thus could serve as a positive control in this study. In the present examination, each compound was evaluated for antimigratory activity at a noncytotoxic concentration (0.1 *μ*g/mL) to avoid the possible interfering cytotoxic effect. The human lung cancer H460 cells were then allowed to migrate in the presence or absence of the test compound for 0, 6, 12, 24, and 48 h, after which the migratory activity was measured. The results, as shown in Figures 2, 3, 4, and 5, indicated that each of the tested compounds exhibited significant antimigratory activity in comparison with the untreated control. As summarized in Figure 6, all of the compounds (**1**,** 3**,** 4**, and** 6**) inhibited the migration of the cells across the wound space in a time-dependent manner. During the first 24 h, dendroflorin (**6**) appeared to be the strongest antimigratory compound; however, after 48 h, moscatilin (**1**) became the most potent compound, as a result of the substantial increase of its activity during the period of 24–48 h. It should be mentioned that the antimigratory effects of moscatilin (**1**) and gigantol (**3**) have been earlier demonstrated in other cancer cells, for example, human lung H23 and breast MDA-MB-231 cells [9, 10, 26]. Additionally, gigantol (**3**) has been shown to suppress lung cancer cell migration through a caveolin-1-dependent pathway [26]. It is worth noting that, among the compounds tested in this study, gigantol (**3**) was the weakest antimigratory agent despite its strong cytotoxicity, suggesting that no linear correlation exists between cytotoxicity and antimigratory activity.

It is known that cell migration may be regulated by the caveolin-1 and/or the protein kinase B (Akt) signaling pathway [27]. In certain cases, particularly in lung cancer migration, overexpression of caveolin-1 is believed to play the predominant role [16]. The apparent high level of caveolin-1 expression in H460 cells in this study (data not shown) might suggest the involvement of caveolin-1 in the cancer cell migration, although more detailed studies are needed before any conclusion can be drawn.

## 4. Conclusion

In summary, in this communication we provide new information on the cytotoxic and antimigratory effects of the phenolic compounds isolated from* Dendrobium brymerianum*. To the best of our knowledge, this is the first report on the biological activities of this plant. Our findings on the antimigratory activities of moscatilin (**1**) and gigantol (**3**) are in agreement with earlier reports. In addition, our preliminary data revealed for the first time the antimigratory potential of lusianthridin (**4**) and dendroflorin (**6**). The biological data obtained in the present study should be useful for the future development of new antimetastatic drugs.

## Supplementary Material

Lyon/Geneva, 12 December 2013 – The International Agency for Research on Cancer (IARC), the specialized cancer agency of the World Health Organization, today released the latest data on cancer incidence, mortality, and prevalence worldwide.1 The new version of IARC's online database, GLOBOCAN 2012, provides the most recent estimates for 28 types of cancer in 184 countries worldwide and offers a comprehensive overview of the global cancer burden.
GLOBOCAN 2012 reveals striking patterns of cancer in women and highlights that priority should be given to cancer prevention and control measures for breast and cervical cancers globally.
Global burden rises to 14.1 million new cases and 8.2 million cancer deaths in 2012
According to GLOBOCAN 2012, an estimated 14.1 million new cancer cases and 8.2 million cancer-related deaths occurred in 2012, compared with 12.7 million and 7.6 million, respectively, in 2008. Prevalence estimates for 2012 show that there were 32.6 million people (over the age of 15 years) alive who had had a cancer diagnosed in the previous five years.
The most commonly diagnosed cancers worldwide were those of the lung (1.8 million, 13.0% of the total), breast (1.7 million, 11.9%), and colorectum (1.4 million, 9.7%). The most common causes of cancer death were cancers of the lung (1.6 million, 19.4% of the total), liver (0.8 million, 9.1%), and stomach (0.7 million, 8.8%).
Projections based on the GLOBOCAN 2012 estimates predict a substantive increase to 19.3 million new cancer cases per year by 2025, due to growth and ageing of the global population. More than half of all cancers (56.8%) and cancer deaths (64.9%) in 2012 occurred in less developed regions of the world, and these proportions will increase further by 2025.

## Figures and Tables

**Table 1:** IC_50_ values for cytotoxic effect of compounds (**1**–**8**) on human lung cancer H460 cells.

Compounds	IC_50_ (*μ*g/mL)
Moscatilin (**1**)	196.7 ± 2.62
Flavanthrinin (**2**)	>200
Gigantol (**3**)	23.4 ± 3.99
Lusianthridin (**4**)	65.0 ± 3.51
Nobilone (**5**)	>200
Dendroflorin (**6**)	125.8 ± 3.29
Denchrysan B (**7**)	>200
Tristin (**8**)	>200
