# Supplementary material for: Cytotoxic and Antimigratory Activities of Phenolic Compounds from Dendrobium brymerianum
Source: Evid Based Complement Alternat Med. 2015 Jan 15;2015:350410. doi: 10.1155/2015/350410 (PMC4312652; doi:10.1155/2015/350410)
Supplement: Supplementary file 1 — Lyon/Geneva, 12 December 2013 – The International Agency for Research on Cancer (IARC), the specialized cancer agency of the World Health Organization, today released the latest data on cancer incidence, mortality, and prevalence worldwide.1 The new version of IARC's online database, GLOBOCAN 2012, provides the most recent estimates for 28 types of cancer in 184 countries worldwide and offers a comprehensive overview of the global cancer burden. GLOBOCAN 2012 reveals striking patterns of cancer in women and highlights that priority should be given to cancer prevention and control measures for breast and cervical cancers globally. Global burden rises to 14.1 million new cases and 8.2 million cancer deaths in 2012 According to GLOBOCAN 2012, an estimated 14.1 million new cancer cases and 8.2 million cancer-related deaths occurred in 2012, compared with 12.7 million and 7.6 million, respectively, in 2008. Prevalence estimates for 2012 show that there were 32.6 million people (over the age of 15 years) alive who had had a cancer diagnosed in the previous five years. The most commonly diagnosed cancers worldwide were those of the lung (1.8 million, 13.0% of the total), breast (1.7 million, 11.9%), and colorectum (1.4 million, 9.7%). The most common causes of cancer death were cancers of the lung (1.6 million, 19.4% of the total), liver (0.8 million, 9.1%), and stomach (0.7 million, 8.8%). Projections based on the GLOBOCAN 2012 estimates predict a substantive increase to 19.3 million new cancer cases per year by 2025, due to growth and ageing of the global population. More than half of all cancers (56.8%) and cancer deaths (64.9%) in 2012 occurred in less developed regions of the world, and these proportions will increase further by 2025. [file 350410.f1.pdf]

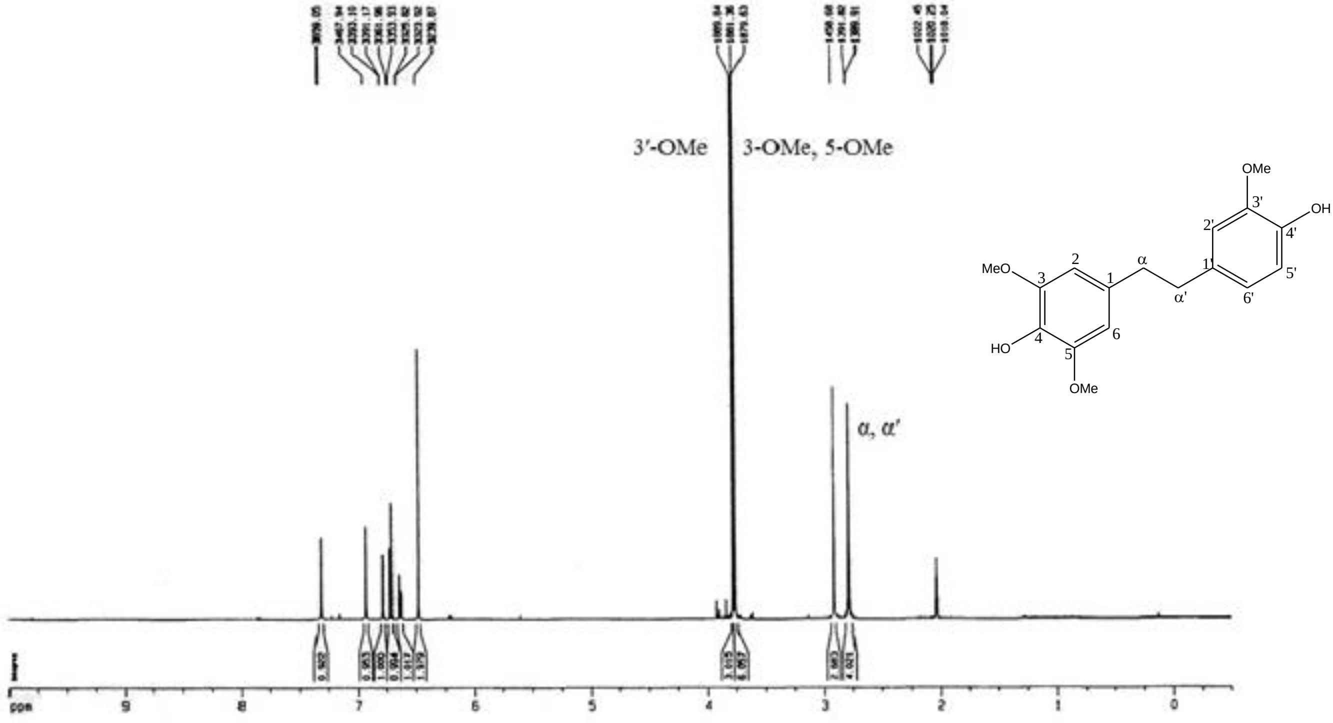

Figure 1 ^{1}H-NMR (500 MHz) spectrum of Moscatilin (acetone-d_6)

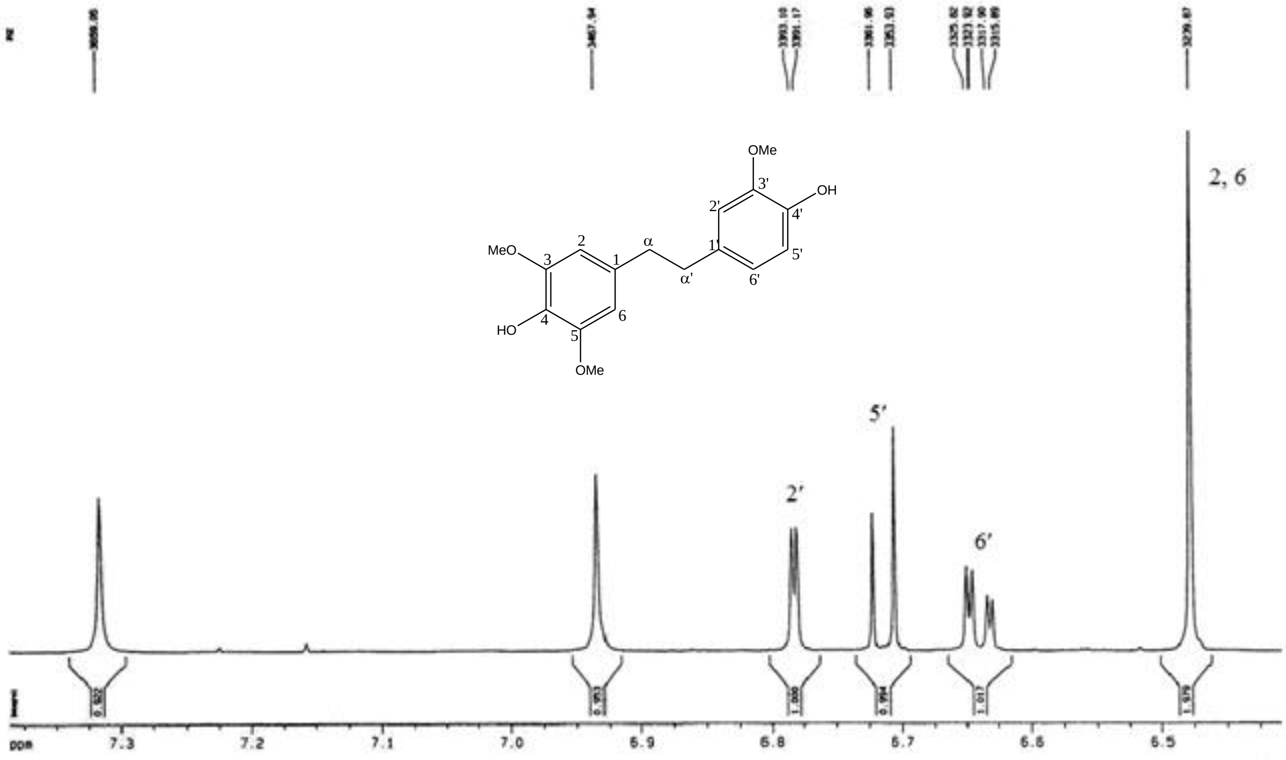

Figure 2 ^{1}H-NMR (500 MHz) spectrum of Moscatilin (acetone-d_6)

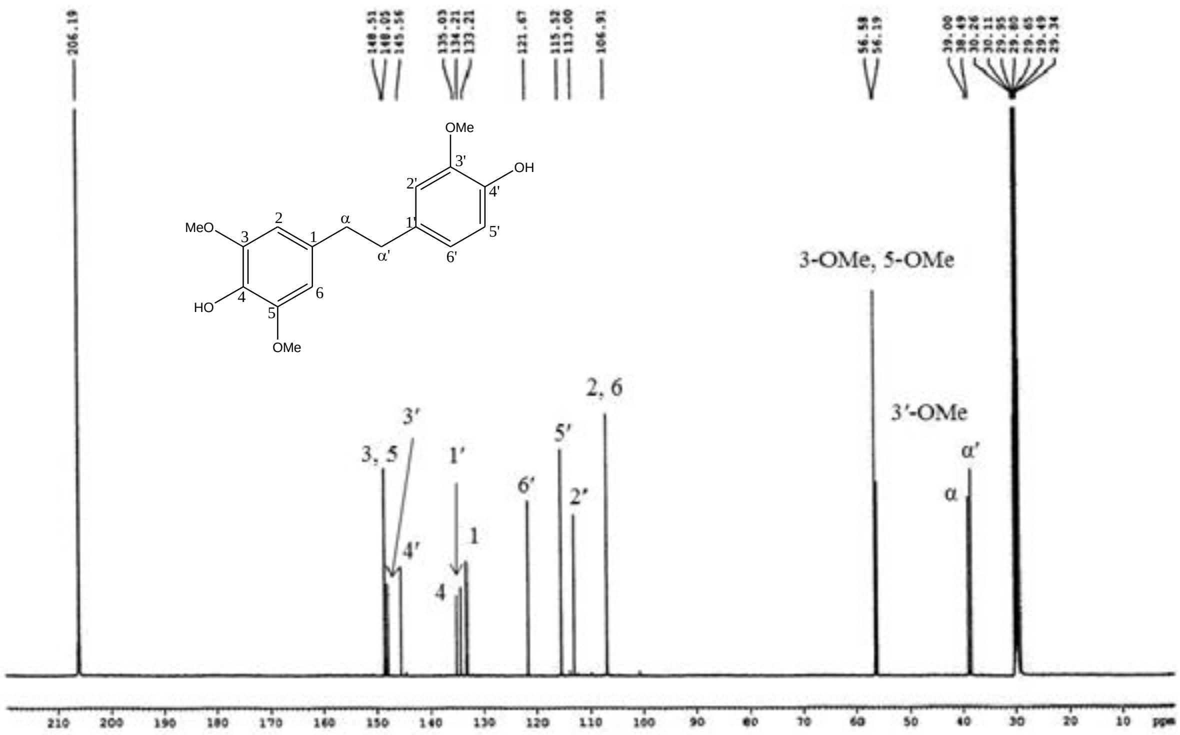

Figure 3 ^{13}C-NMR (125 MHz) spectrum of Moscatilin (acetone-d_6)

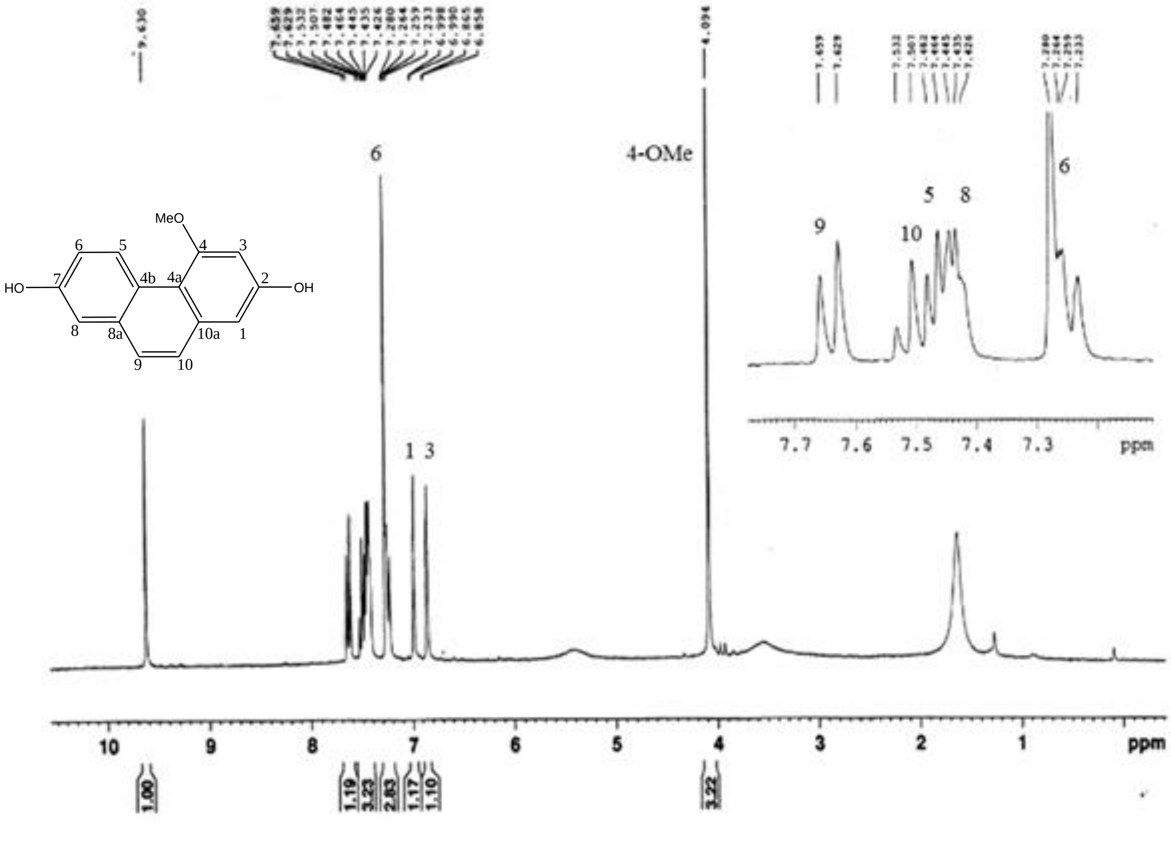

Figure 4 ^{1}H-NMR (300 MHz) spectrum of Flavanthrinin ($CDCl_3$)

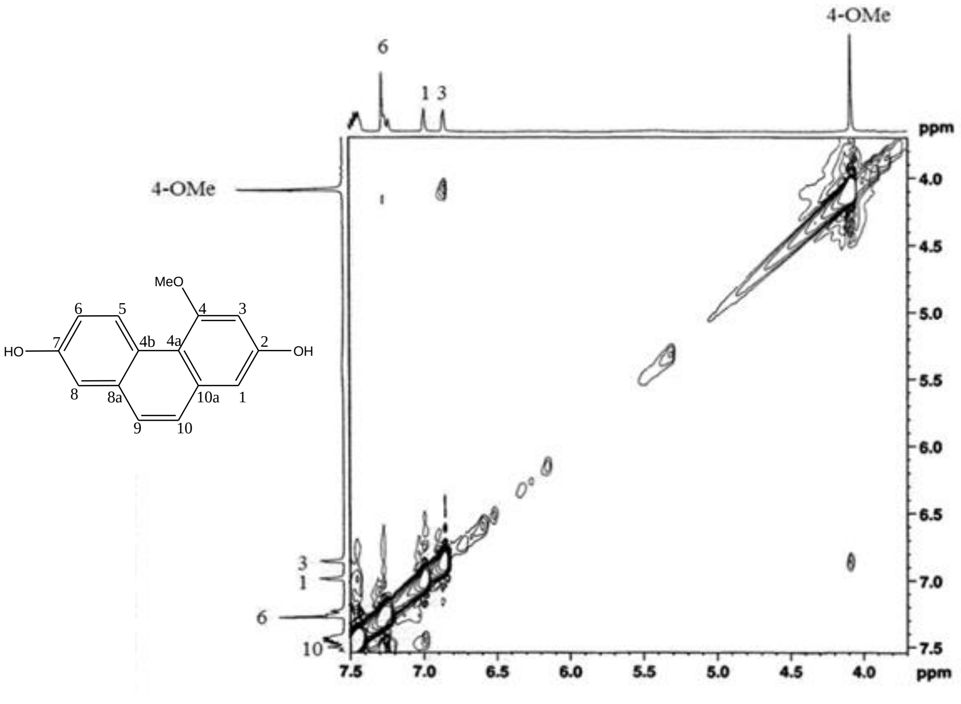

Figure 5 NOSEY spectrum of Flavanthrinin

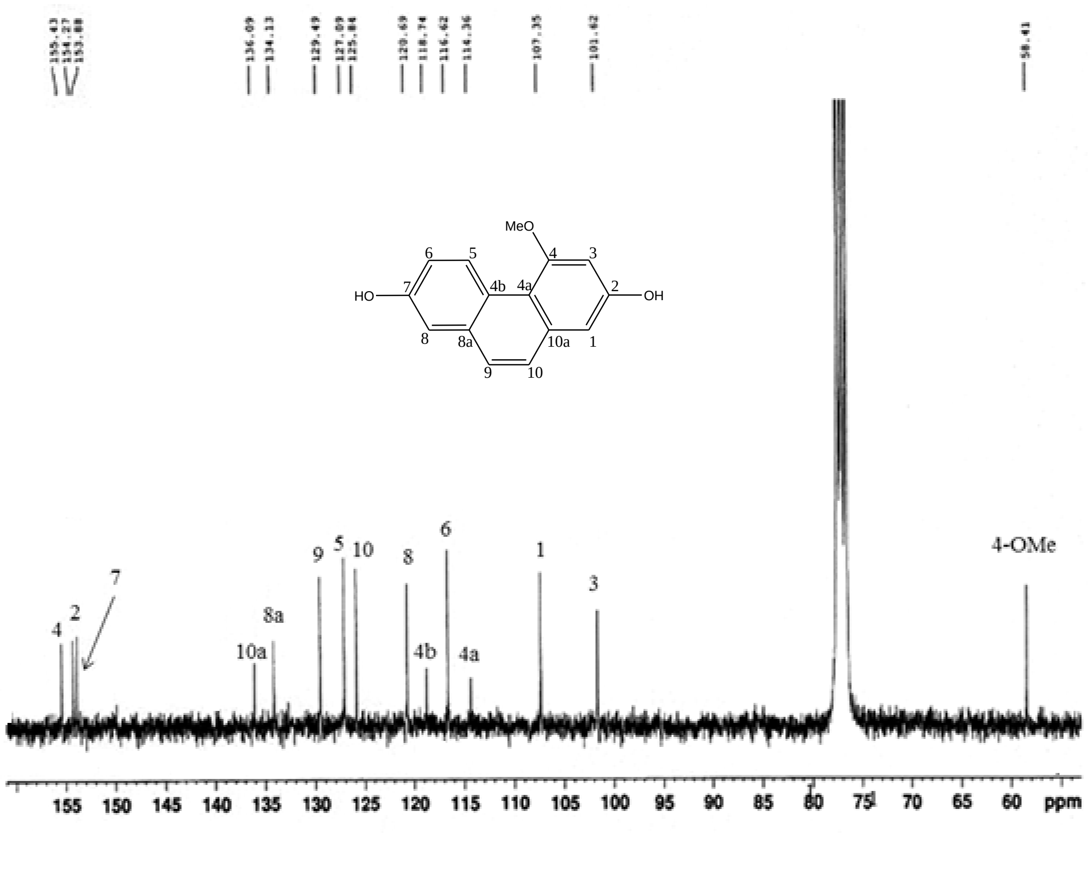

Figure 6 ^{13}C-NMR (75 MHz) spectrum of Flavanthrinin ($CDCl_3$)

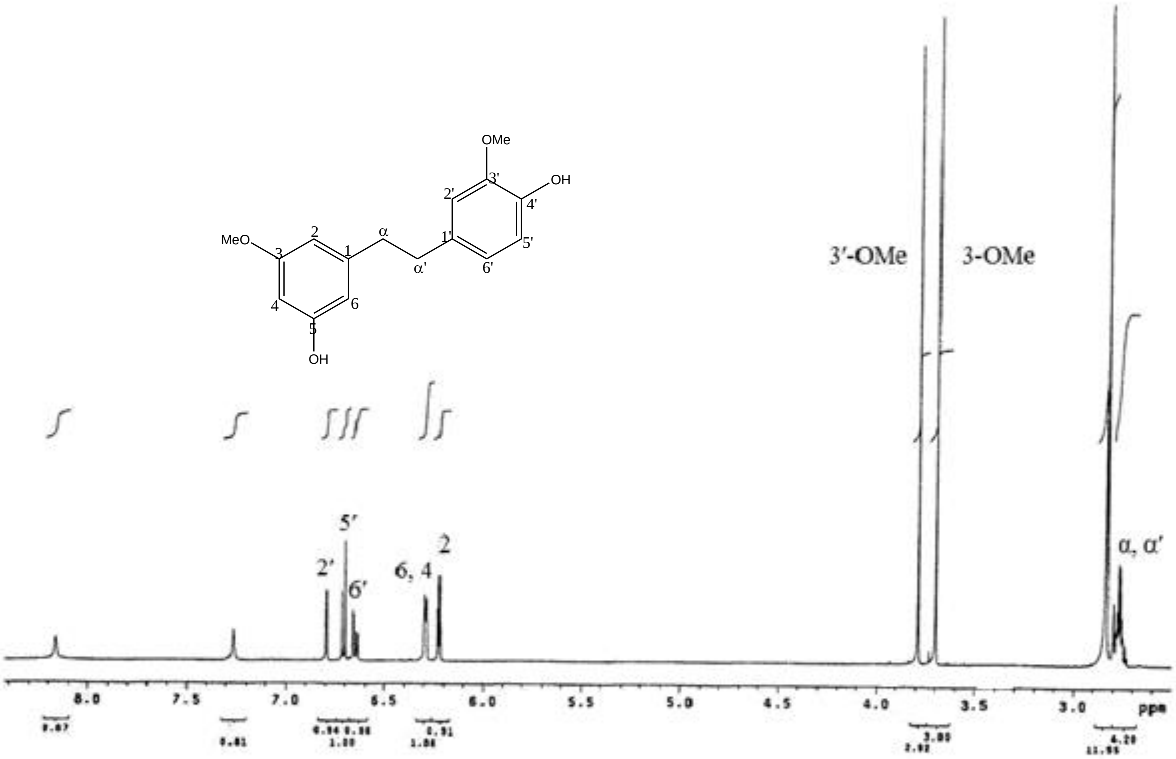

Figure 7 ^{1}H-NMR (500 MHz) spectrum of Gigantol (acetone-d_6)

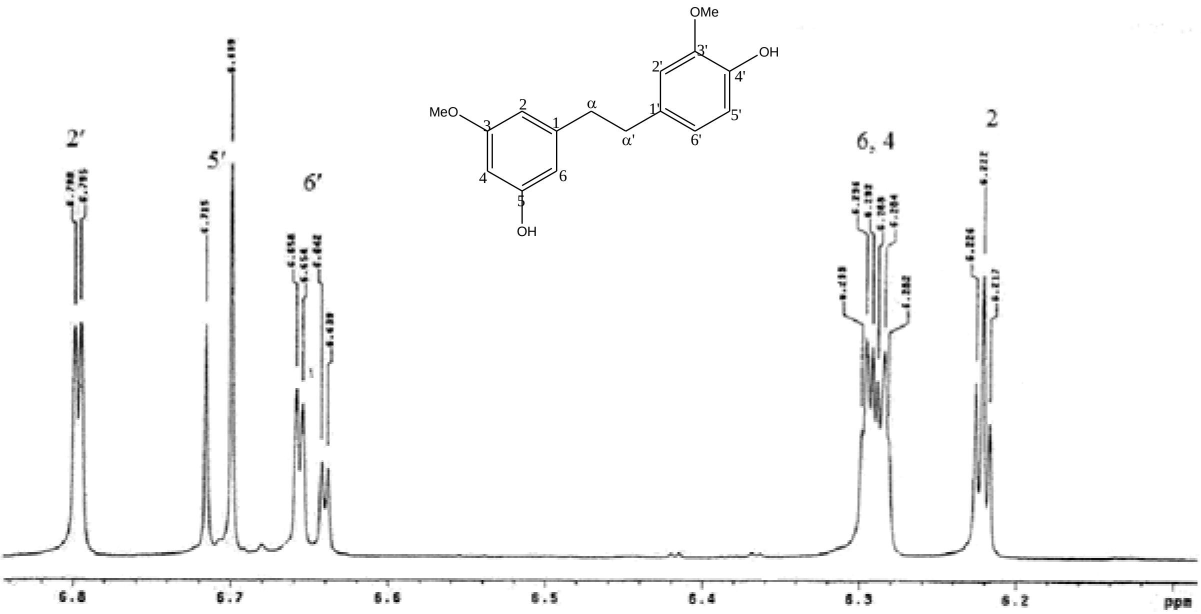

Figure 8 ^{1}H-NMR (500 MHz) spectrum of Gigantol (acetone-d_6)

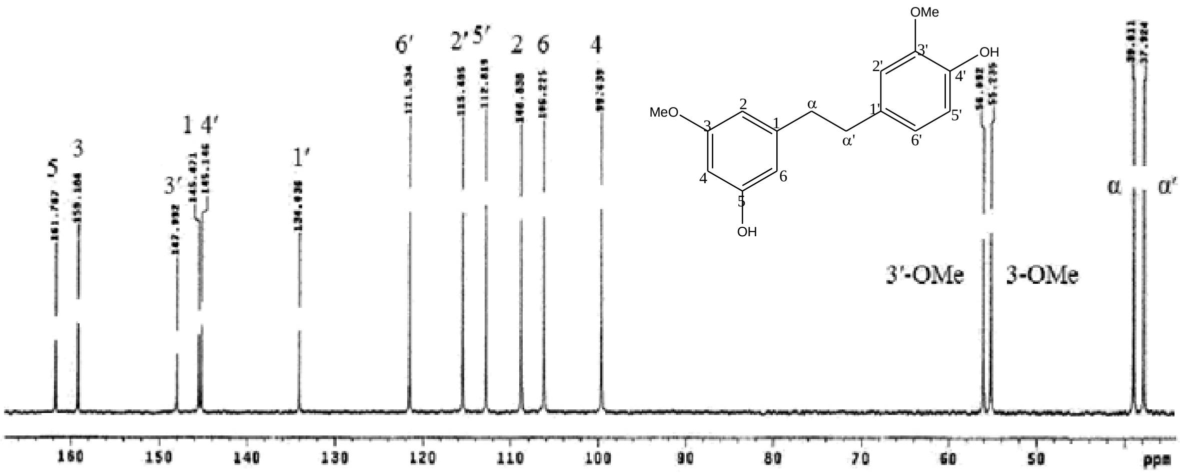

Figure 9 ^{13}C-NMR (125 MHz) spectrum of Gigantol (acetone-*d_6*)

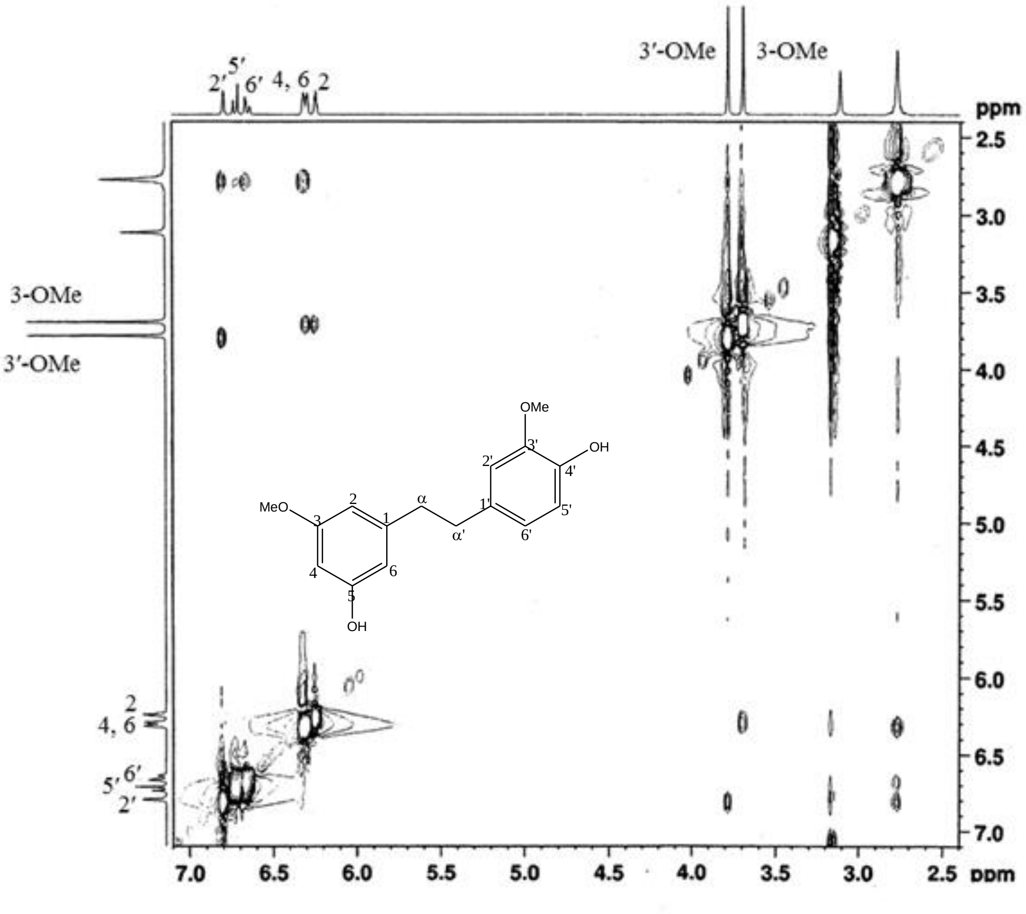

Figure 10 NOESY spectrum of compound Gigantol (acetone-d_6)

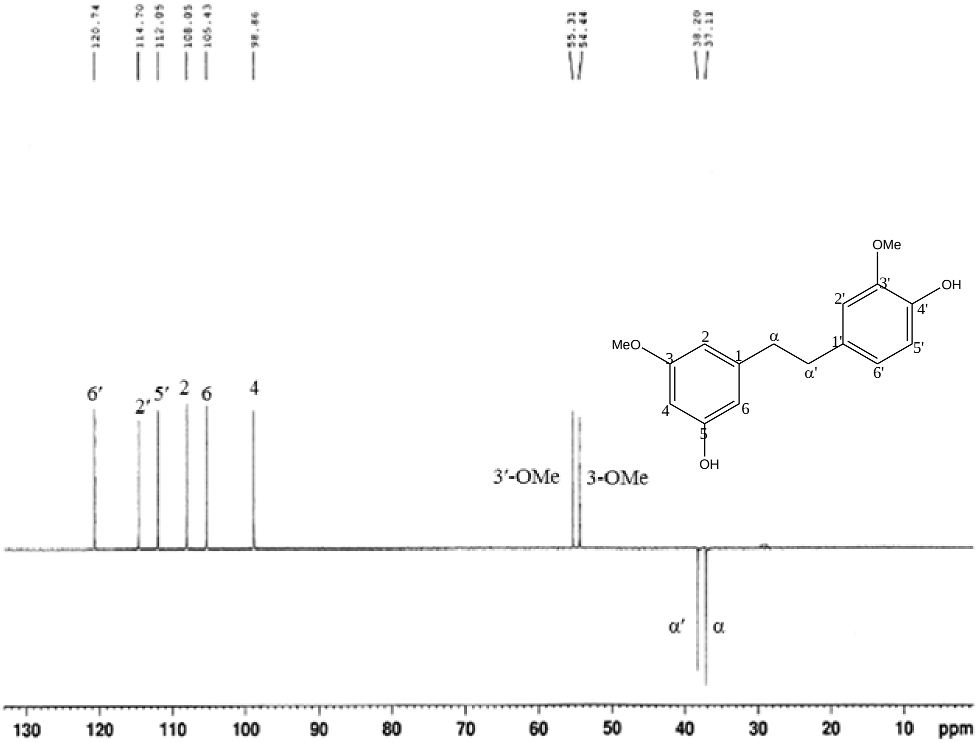

Figure 11 DEPT 135 spectrum of Gigantol (acetone-d_6)

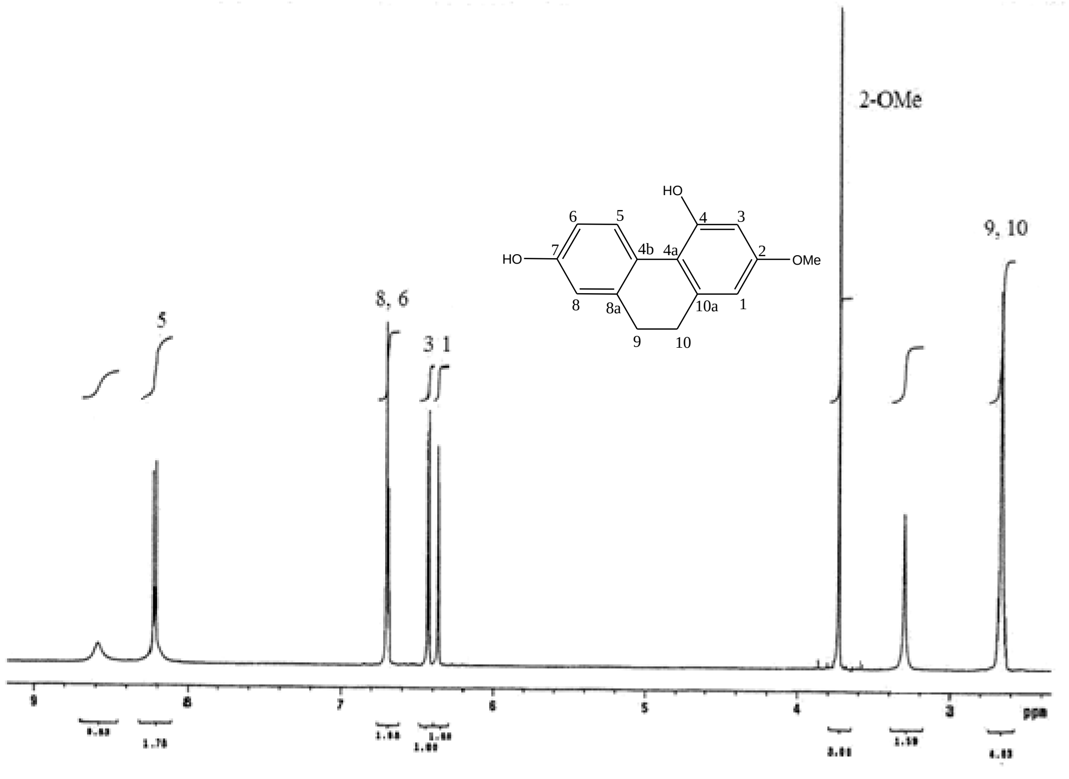

Figure 12 ^{1}H-NMR (500 MHz) spectrum of Lusianthridin (acetone-d_6)

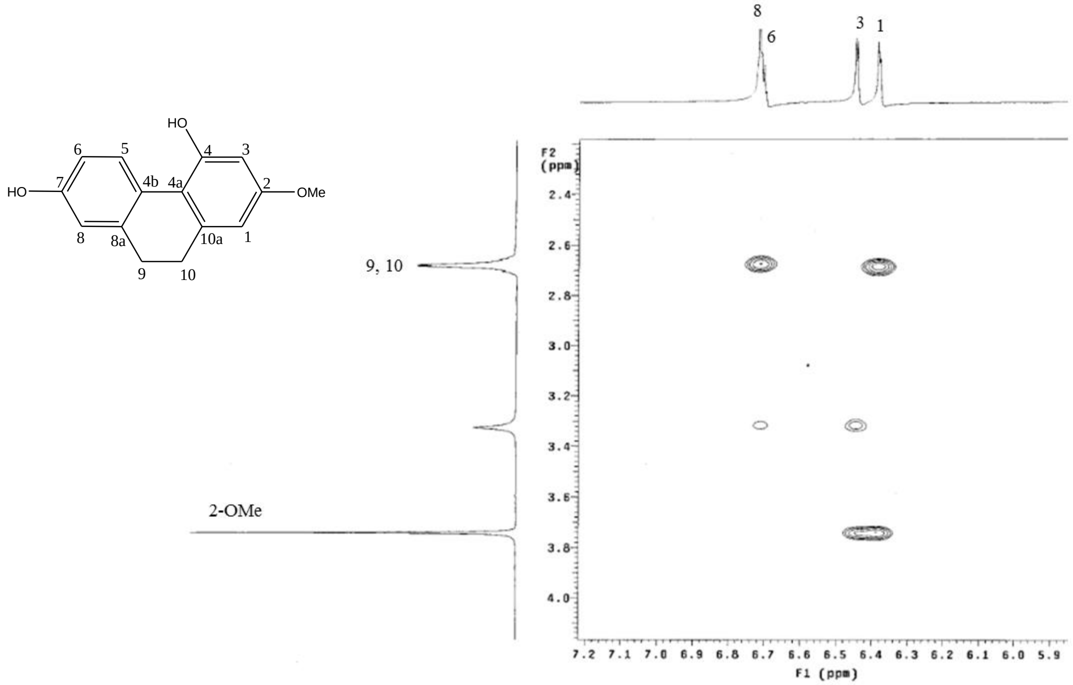

Figure 13 NOESY spectrum of Lusianthridin (acetone-d_6)

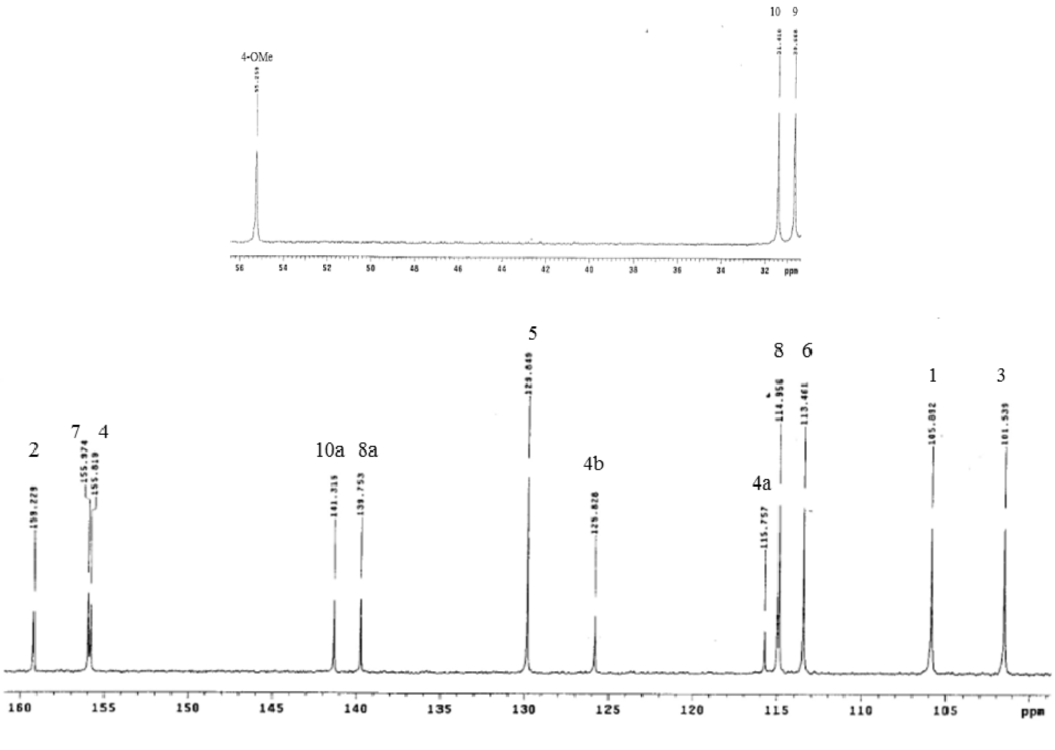

Figure 14 ^{13}C-NMR (125 MHz) spectrum of Lusianthridin (acetone-d_6)

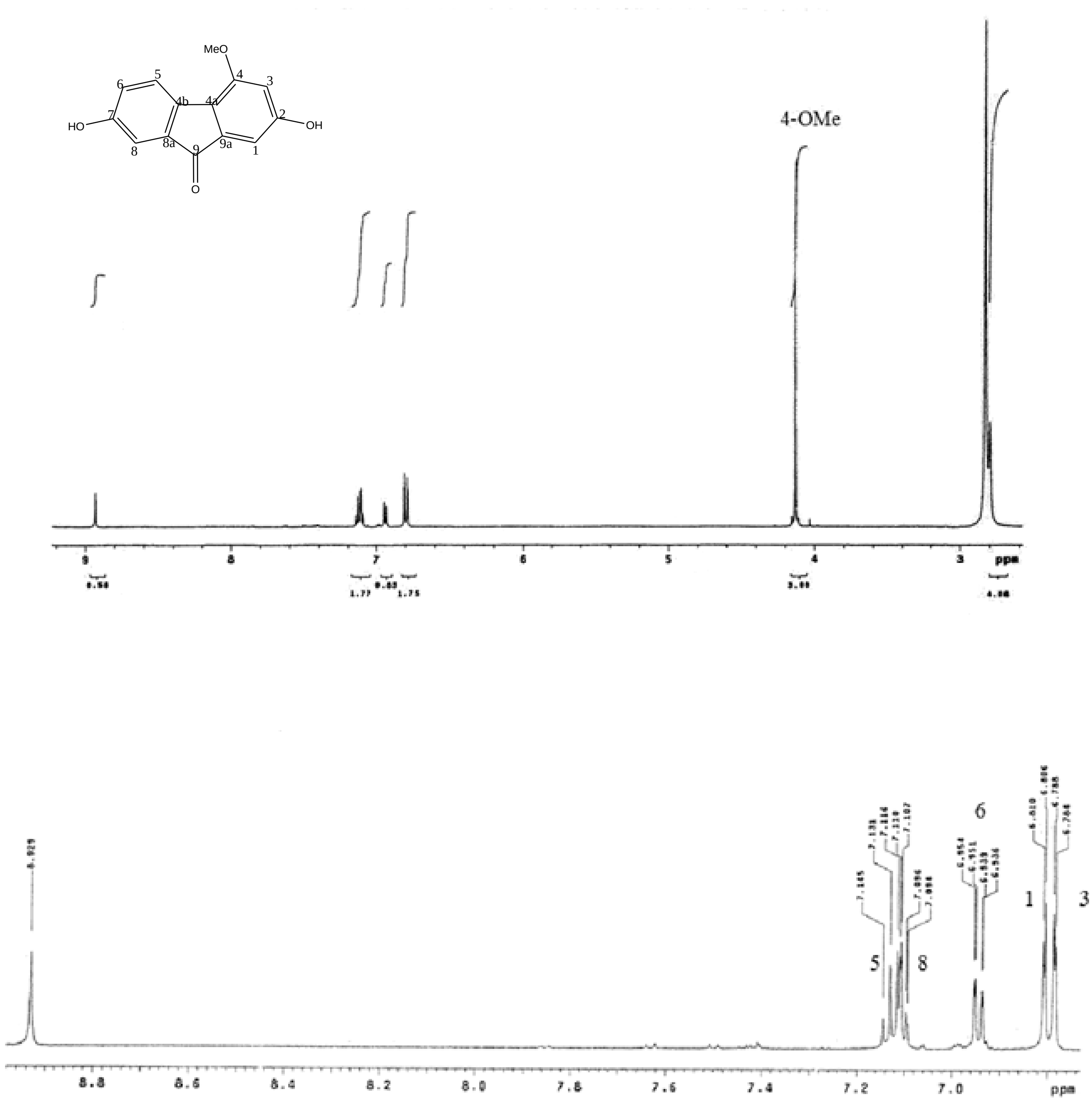

Figure 15 ^{1}H-NMR (500 MHz) spectrum of Nobilone (acetone-d_6)

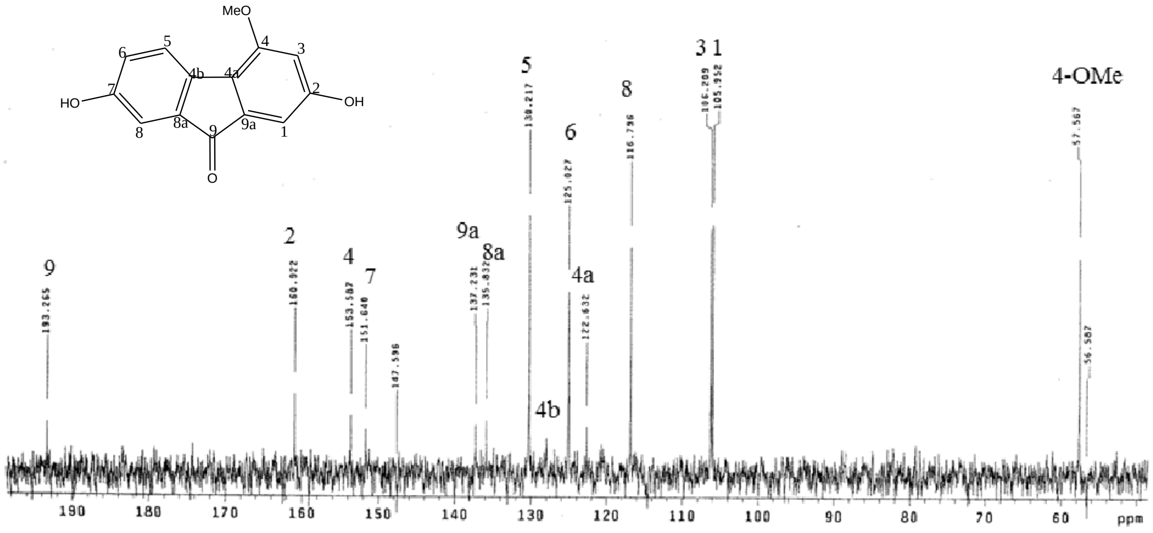

Figure 16 ^{13}C-NMR (125 MHz) spectrum of Nobilone (acetone-d_6)

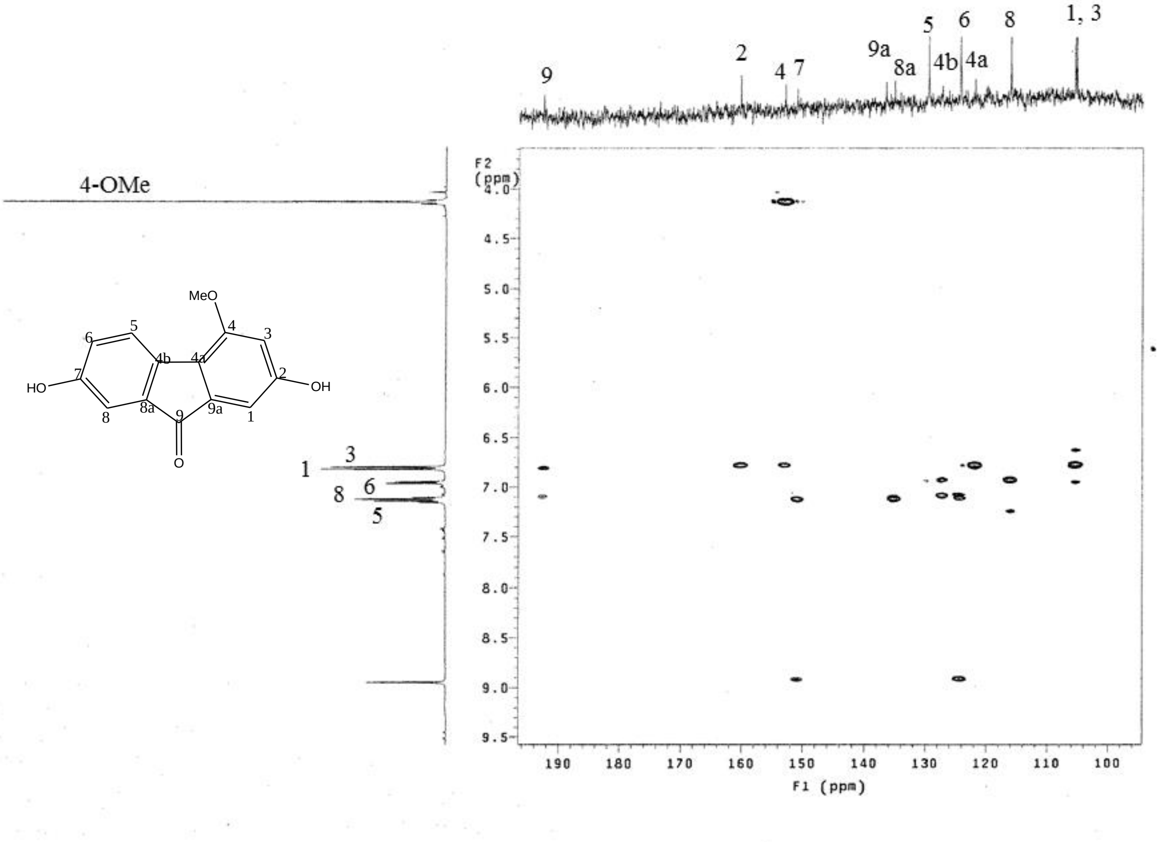

Figure 17 HMBC spectrum of Nobilone (acetone-d_6)

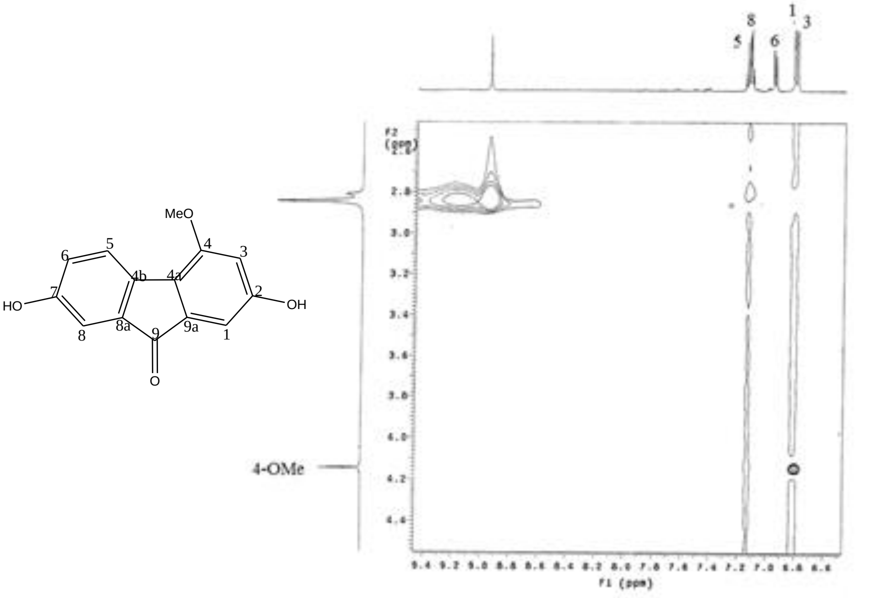

Figure 18 NOESY spectrum of Nobilone (acetone-d_6)

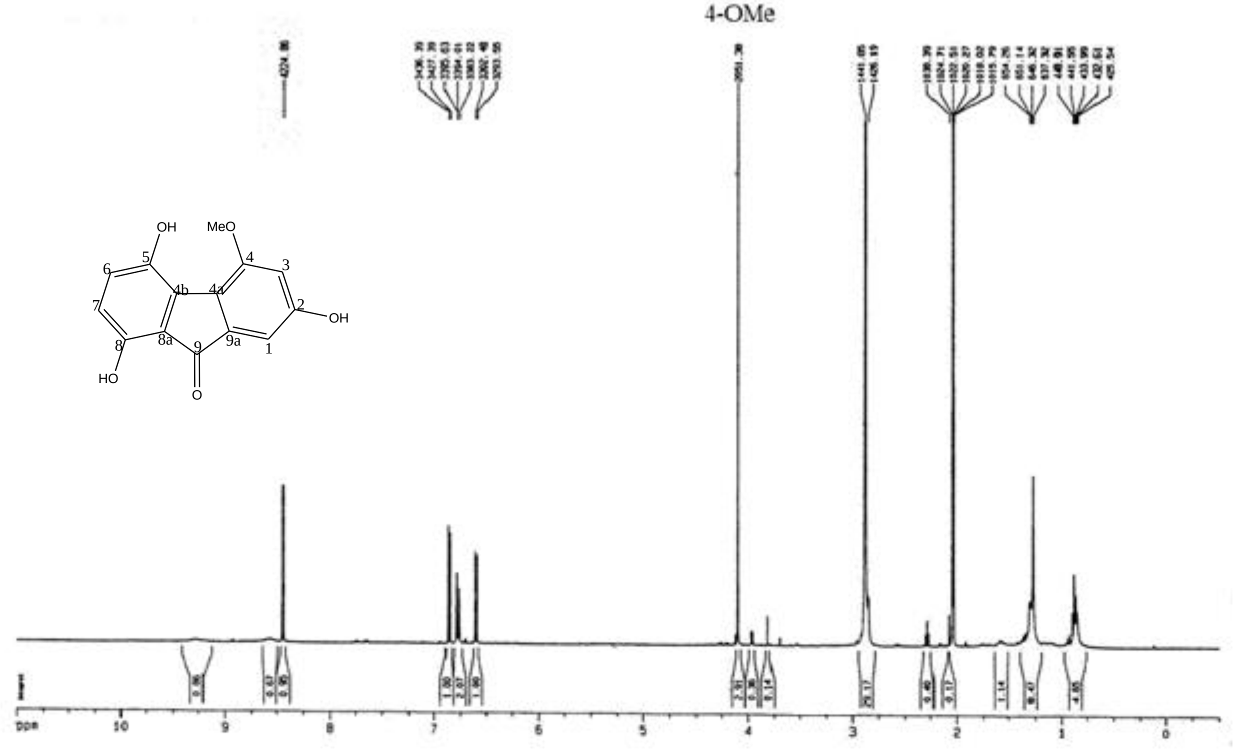

Figure 19 ^{1}H-NMR (500 MHz) spectrum of Dendroflorin (acetone-d_6)

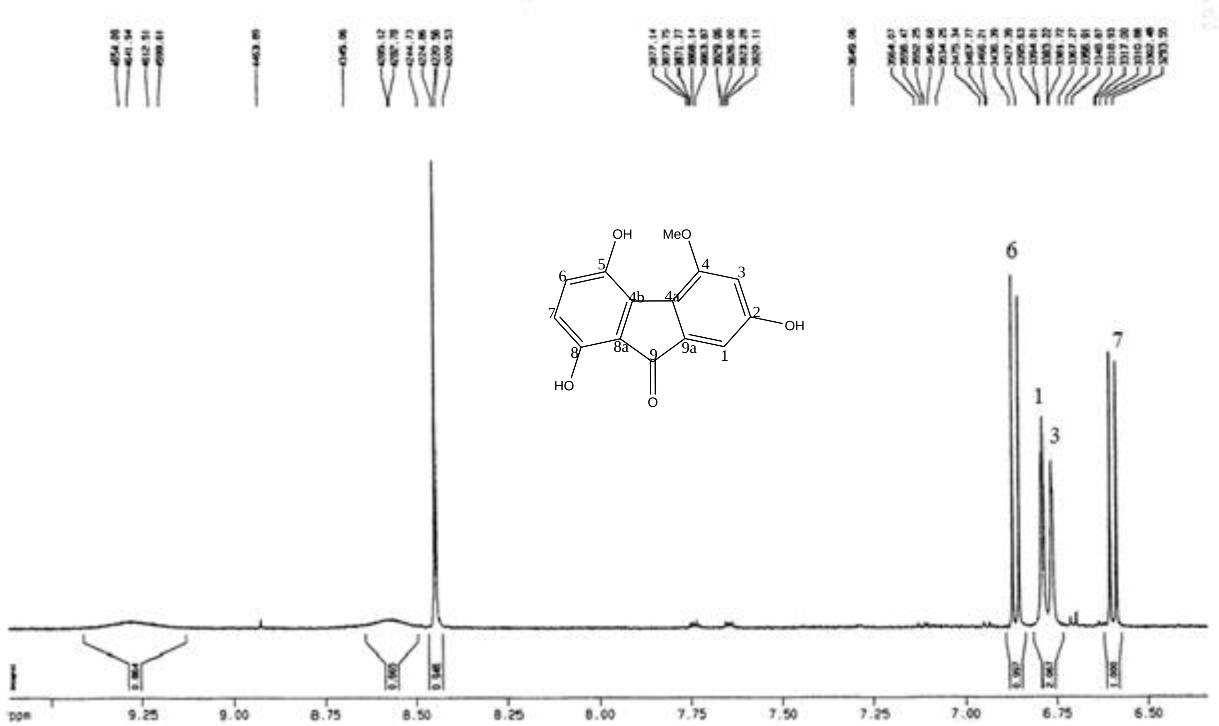

Figure 20 ^{1}H-NMR (500 MHz) spectrum of Dendroflorin (acetone-d_6)

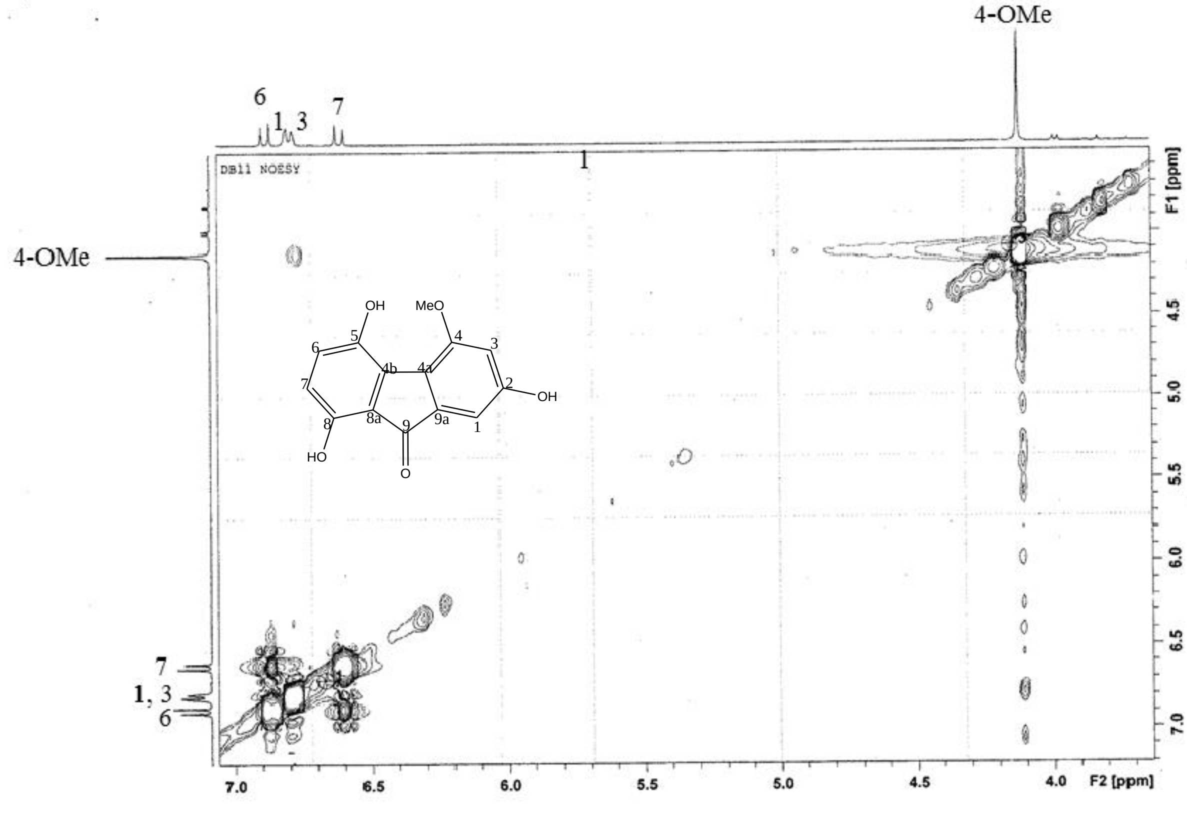

Figure 21 NOESY spectrum of Dendroflorin (acetone-d_6)

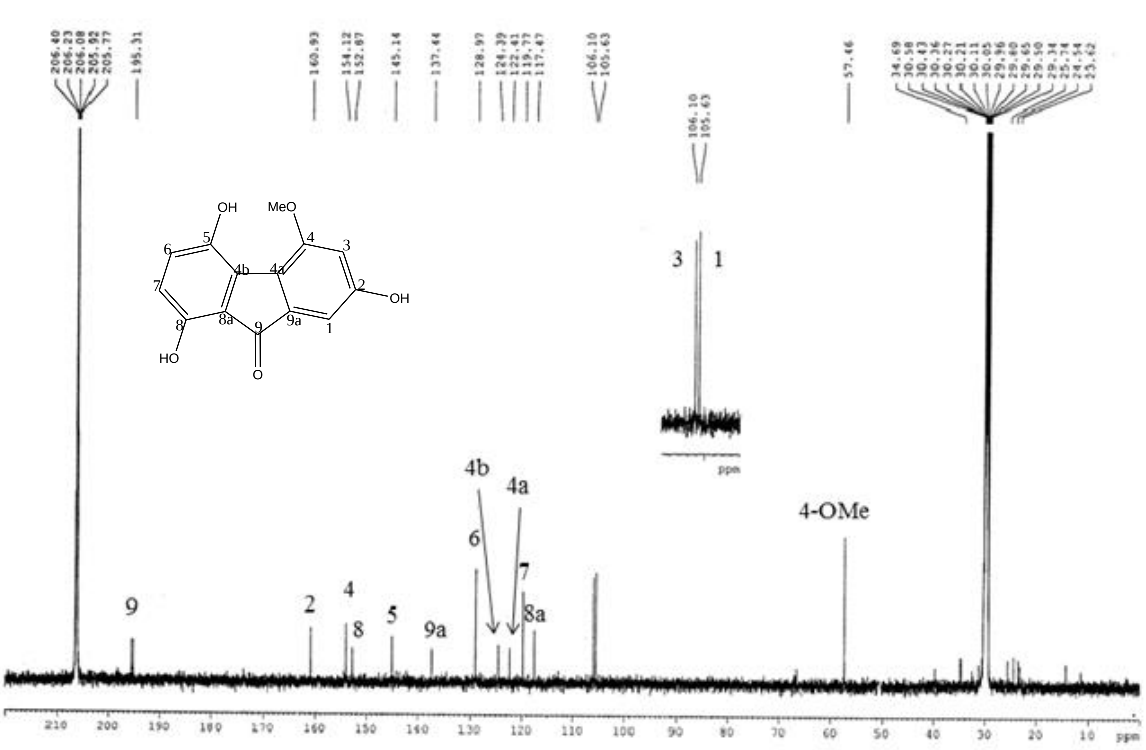

Figure 22 ^{13}C-NMR (125 MHz) spectrum of Dendroflorin (acetone-d_6)

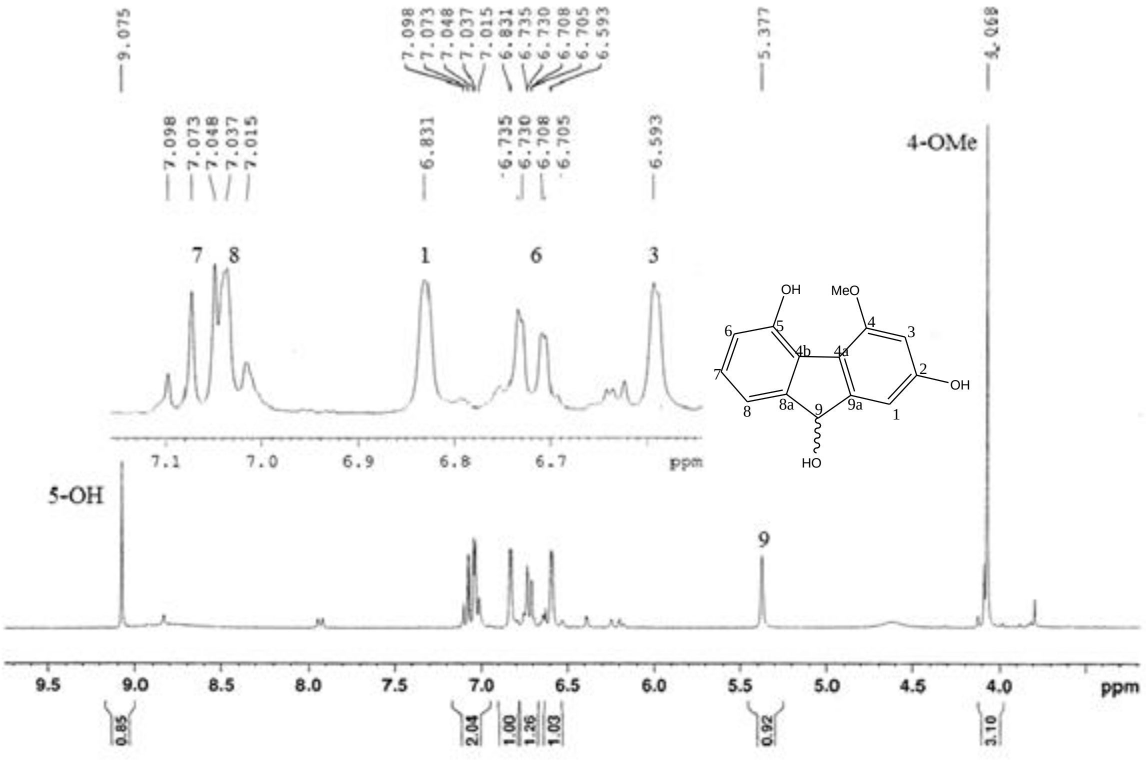

Figure 23 ^{1}H-NMR (300 MHz) spectrum of Denchrysan B (acetone-d_6)

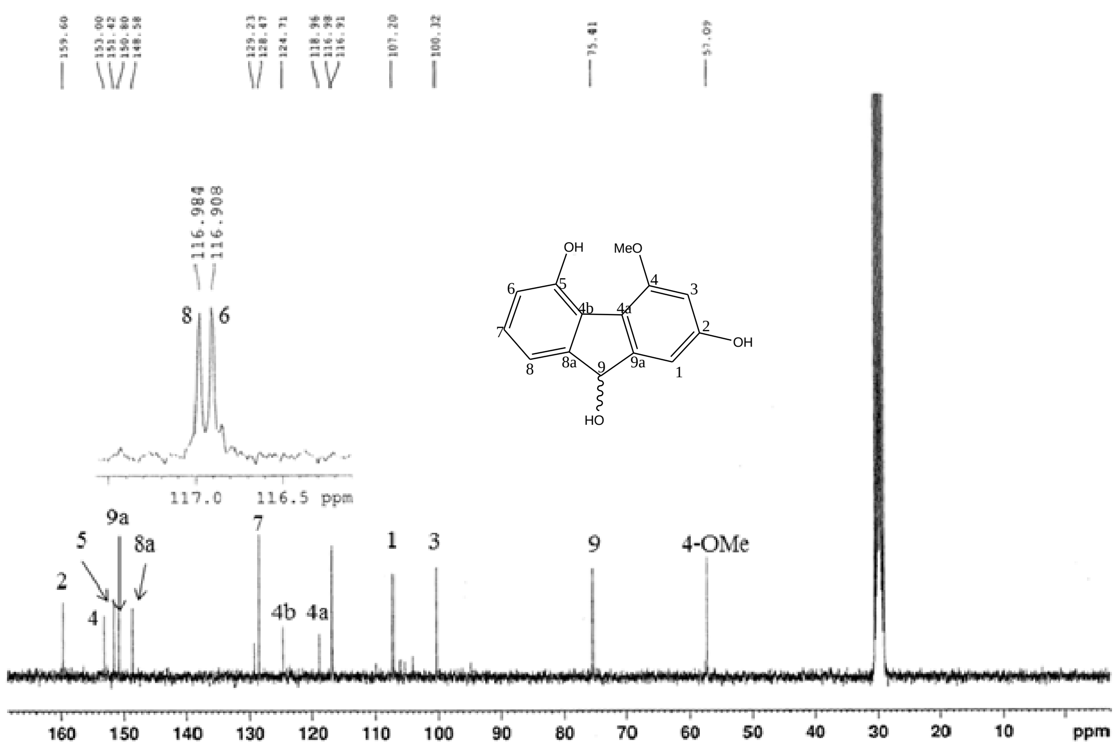

Figure 24 ^{13}C-NMR (75 MHz) spectrum of Denchrysan B (acetone-d_6)

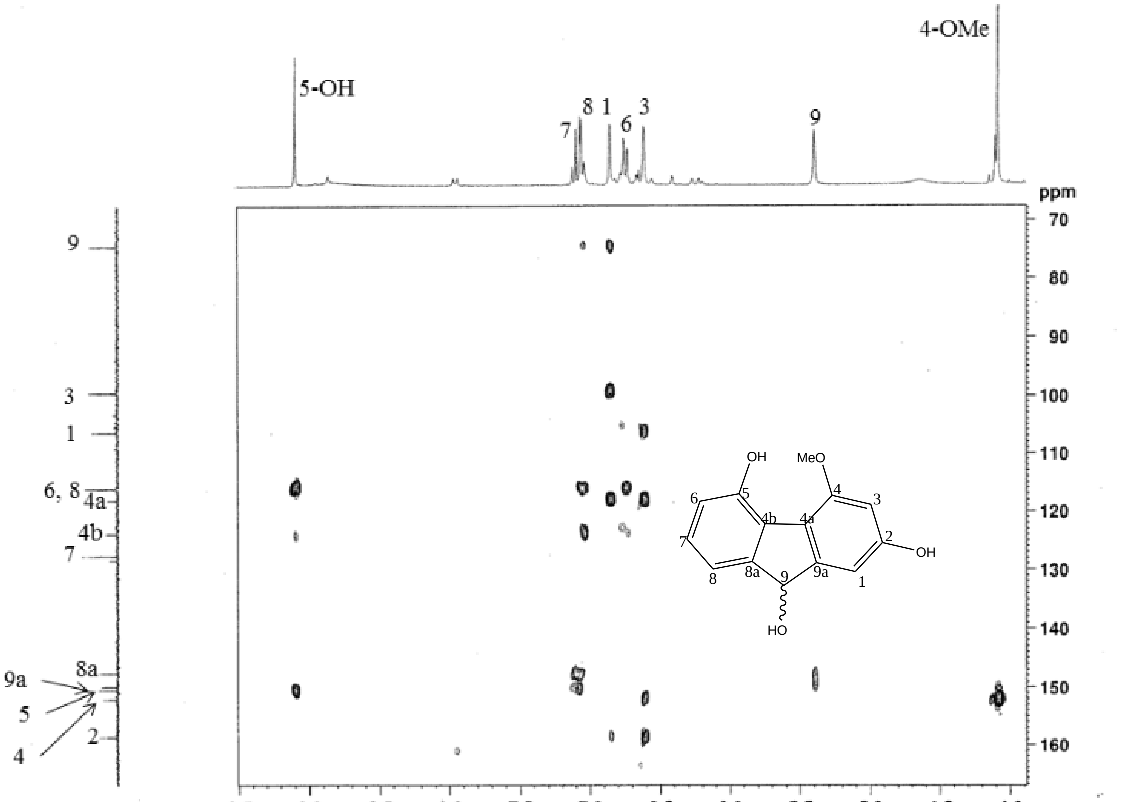

Figure 25 HMBC spectrum of Denchrysan B (acetone-d_6)

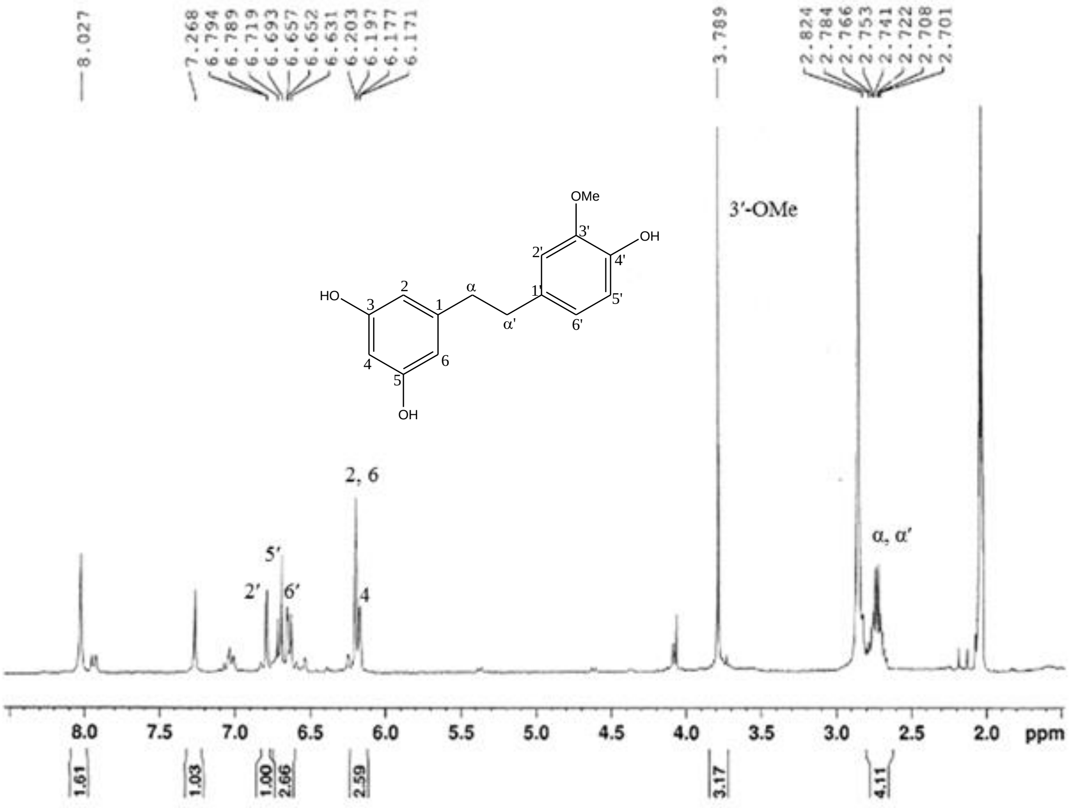

Figure 26 ^{1}H-NMR (300 MHz) spectrum of Tristin (acetone-d_6)

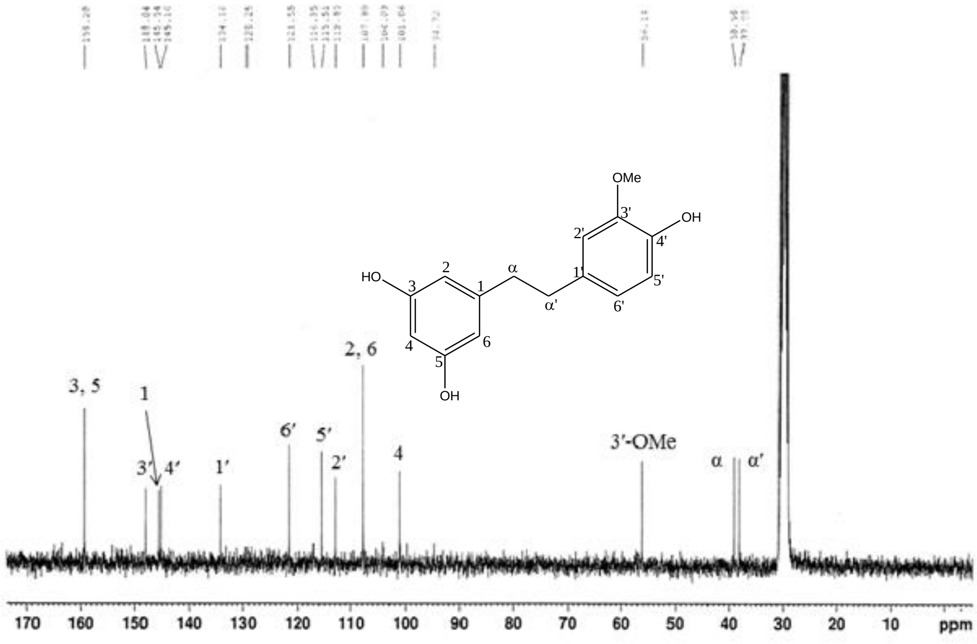

Figure 27 ^{13}C-NMR (75 MHz) spectrum of Tristin (acetone-d_6)

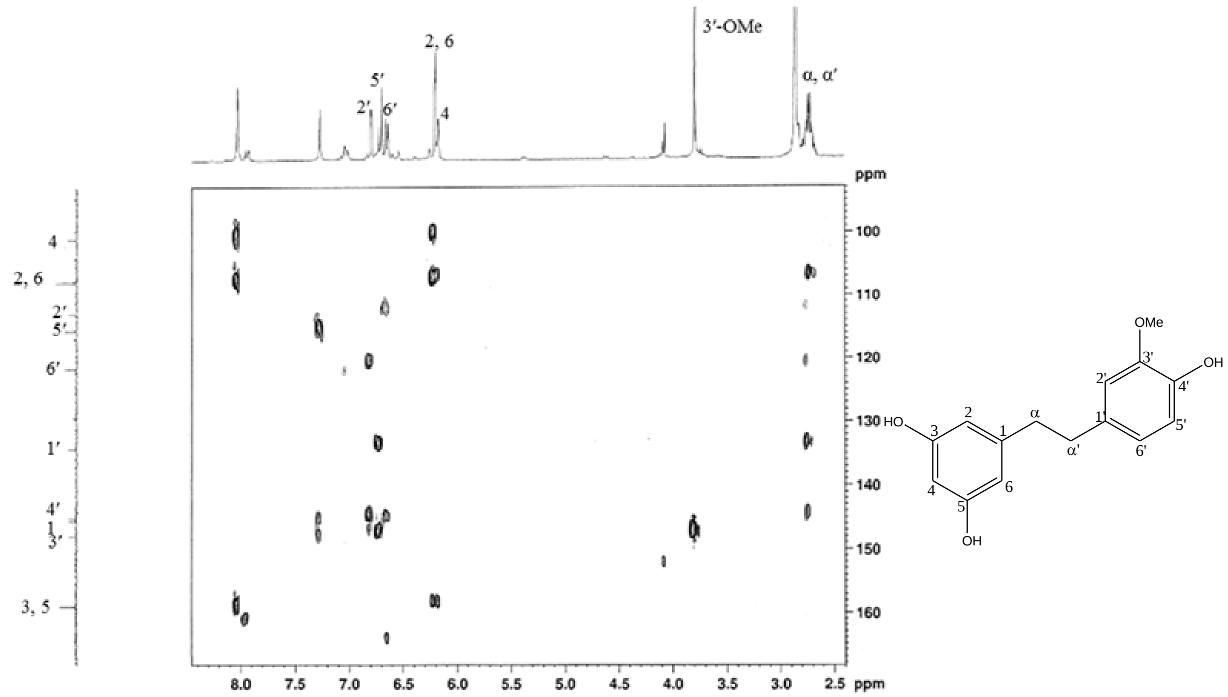

Figure 28 HMBC spectrum of Tristin (acetone-d_6)

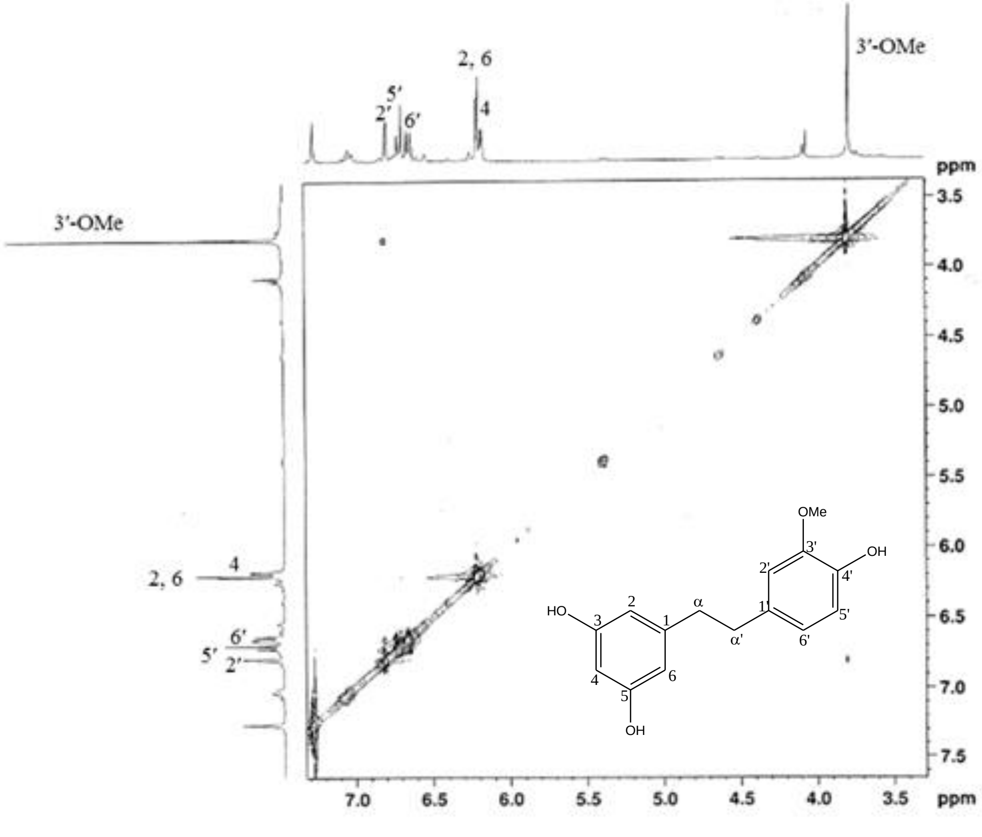

Figure 29 NOESY spectrum of Tristin (acetone-d_6)
